# The Impact of Angiotensin-Converting Enzyme Inhibitors or Angiotensin II Receptor Blockers on Clinical Outcomes of Acute Kidney Disease Patients: A Systematic Review and Meta-Analysis

**DOI:** 10.3389/fphar.2021.665250

**Published:** 2021-07-20

**Authors:** Jui-Yi Chen, I-Jung Tsai, Heng-Chih Pan, Hung-Wei Liao, Javier A. Neyra, Vin-Cent Wu, Jeff S. Chueh

**Affiliations:** ^1^ Division of Nephrology, Chi Mei Medical Center, Department of Internal Medicine, Tainan, Taiwan; ^2^ Division of Nephrology, Department of Pediatrics, National Taiwan University Children’s Hospital, Taipei, Taiwan; ^3^ College of Medicine, Graduate Institute of Clinical Medicine, National Taiwan University, Taipei, Taiwan; ^4^ Division of Nephrology, Keelung Chang Gung Memorial Hospital, Department of Internal Medicine, Taipei, Taiwan; ^5^ Chinru Clinic, Taipei, Taiwan; ^6^ Division of Nephrology, Department of Internal Medicine, Bone and Mineral Metabolism, University of Kentucky, Lexington, KY, United States; ^7^ Department of Internal Medicine, National Taiwan University Hospital, Taipei, Taiwan; ^8^ NSARF (National Taiwan University Hospital Study Group of ARF) and TAIPAI (Taiwan Primary Aldosteronism Investigators), Taipei, Taiwan; ^9^ Cleveland Clinic, Cleveland Clinic Lerner College of Medicine, Glickman Urological and Kidney Institute, Cleveland, OH, United States; ^10^ Department of Urology, College of Medicine, National Taiwan University Hospital, National Taiwan University, Taipei, Taiwan

**Keywords:** angiotensin-converting enzyme inhibitor, angiotensin II receptor blocker, acute kidney disease, RAAS, chronic kidney disease, post-AKI care, dialysis, outcome

## Abstract

**Background:** Acute kidney injury (AKI) may increase the risk of chronic kidney disease (CKD), development of end-stage renal disease (ESRD), and mortality. However, the impact of exposure to angiotensin-converting enzyme inhibitor or angiotensin II receptor blocker (ACEi/ARB) in patients experiencing AKI/acute kidney disease (AKD) is still unclear.

**Methods:** In this systematic review, we searched all relevant studies from PubMed, Embase, Cochrane, Medline, Collaboration Central Register of Controlled Clinical Trials, Cochrane Systematic Reviews, and ClinicalTrials.gov until July 21, 2020. We evaluated whether the exposure to ACEi/ARB after AKI onset alters recovery paths of AKD and impacts risks of all-cause mortality, recurrent AKI, or incident CKD. We rated the certainty of evidence according to Cochrane methods and the GRADE approach.

**Results:** A total of seven articles, involving 70,801 patients, were included in this meta-analysis. The overall patient mortality rate in this meta-analysis was 28.4%. Among AKI patients, all-cause mortality was lower in ACEi/ARB users than in ACEi/ARB nonusers (log odds ratio (OR) −0.37, 95% confidence interval (CI): −0.42–−0.32, p < 0.01). The risk of recurrent adverse kidney events after AKI was lower in ACEi/ARB users than in nonusers (logOR −0.25, 95% CI: −0.33–−0.18, p < 0.01). The risk of hyperkalemia was higher in ACEi/ARB users than in nonusers (logOR 0.43, 95% CI: 0.27–0.59, p < 0.01). Patients with continued use of ACEi/ARB after AKI also had lower mortality risk than those prior ACEi/ARB users but who did not resume ACEi/ARB during AKD (logOR −0.36, 95% CI: −0.4–−0.31, p < 0.01).

**Conclusions:** Exposure to ACEi/ARB after AKI is associated with lower risks of all-cause mortality, recurrent AKI, and progression to incident CKD. Patients with AKI may have a survival benefit by continued use of ACEi/ARB; however, a higher incidence of hyperkalemia associated with ACEi/ARB usage among these patients deserves close clinical monitoring.

## Introduction

Acute kidney injury (AKI) is a frequent event during hospitalization, and it increases the risk of chronic kidney disease (CKD) and end-stage kidney disease (ESKD) in survivors (Heung et al., 2016). Survivors of AKI are also at increased risk of hypertension and cardiovascular disease morbidity and mortality (Parr and Siew, 2016). In CKD patients, many studies have shown that controlling blood pressure effectively with angiotensin-converting enzyme inhibitor and/or angiotensin II receptor blocker (ACEi/ARB) could decrease the risk of dialysis dependence and protect the cardiovascular function (Wang et al., 2018). From the pathophysiologic perspective, AKI could erratically activate the renin-angiotensin-aldosterone system (RAAS), could promote organ fibrosis, and may lead to the development of CKD and cardiac injury. In a cohort study, survivors of AKI had a 22% increase in the odds of developing hypertension (Hsu et al., 2016), implicating the importance of hypertensive care after AKI. Further, the use of ACEi/ARB may prevent heart failure hospitalization after AKI, which contributes to the morbidity of these patients (Silver et al., 2017; Go et al., 2018). In a rat ischemia-reperfusion model, renal blood/plasma flow and glomerular filtration rate (GFR) improved with an ARB, valsartan (Lopau et al., 2001). Thus, ACEi/ARB is considered renal-protective and may improve long-term outcomes after AKI (Gayat et al., 2018).

However, ACEi/ARB usage could also be associated with functional AKI, especially in the setting of acute hypovolemia due to its effect on intrarenal hemodynamics of efferent arteriolar vasodilation and decreased GFR (Khwaja, 2012; Ftouh and Thomas, 2013; Lapi et al., 2013; Brar et al., 2018). Therefore, it has been observed clinically and postulated that ACEi/ARB usage during or shortly after AKI might aggravate further deterioration of kidney function (Guidi et al., 2002). Observational data revealed that using RAAS inhibitors peri-/post-AKI was associated with lower mortality, but higher risk of hospitalization for kidney-related events (Ftouh and Thomas, 2013).

The Kidney Disease, Improving Global Outcomes (KDIGO) reports conducting studies to guide timing of ACEi/ARBs discontinuation and reinitiation in AKI/AKD in different clinical contexts. AKD is defined as the duration of persistent AKI and its recovery phase beyond seven days of AKI onset and up to 90 days (Ostermann et al., 2020). Despite the fact that there have been several elegant studies completed over the last few years (Brar et al., 2018; Gayat et al., 2018; Scarton et al., 2019; Bidulka et al., 2020; Hines et al., 2020; Hsu et al., 2020; Qiao et al., 2020), the impact of ACEi/ARB in the context of AKI/AKD has not been undisputedly confirmed.

Therefore, this study aimed to investigate the impact of exposure on RAAS inhibitors (specifically focusing on ACEi and/or ARB, expressed as ACEi/ARB afterward) after AKI on mortality, recurrent AKI, or incident CKD. A systematic review of the related literature and meta-analysis was performed to provide comprehensive evidence of the effect of ACEi/ARB on selected outcomes of interest.

## Methods

### Search Strategy and Selection Criteria

We reported the meta-analysis according to the Preferred Reporting Items of Systematic Reviews and Meta-Analyses (PRISMA) statement (Higgins et al., 2011) and used Cochrane methods (Salguero et al., 2008). We prospectively submitted the systematic review protocol for registration on PROSPERO (CRD42020204885).

### Study Search Strategy and Selection

The related studies written by all languages were obtained through PubMed, Embase, Cochrane, Medline, Collaboration Central Register of Controlled Clinical Trials, Cochrane Systematic Reviews, and ClinicalTrials.gov until July 21, 2020. The following search terms were used: “Angiotensin converting enzyme Inhibitors”, “Angiotensin receptor blocker,” “Renin angiotensin aldosterone system blockers,” “Renin-angiotensin system blockade,” “After acute kidney injury,” “Acute kidney injury,” “Acute renal failure,” “Advanced kidney disease,” “Mortality,” and “Death.” We included prospective and retrospective cohort studies and observational studies, but case reports and case series were excluded. Two investigators (JY Chen and IJ Tsai) searched and checked all articles separately to prevent bias. If they disagreed on the inclusion of an article, a third author (HW Liao) resolved the dispute. Eligible published studies compared exposure to ACEi/ARB to a control group without the exposure after AKI onset for the risk of mortality, recurrent AKI or worsening kidney function, and progression to CKD.

### Data Extraction and Outcome Assessment

The following data were extracted from the full-text articles: the first author name, year of publication, sample size, study design, patient inclusion/exclusion criteria, patient demographics, clinical outcome, and adverse events (e.g., hyperkalemia). The primary outcome was mortality and the secondary outcome was adverse kidney events, including recurrence of AKI episode(s) or AKD (Brar et al., 2018; Bidulka et al., 2020; Hines et al., 2020; Hsu et al., 2020), sustained doubling of serum creatinine (sCr) concentration (Brar et al., 2018), and worsening kidney function or ESKD (Brar et al., 2018; Qiao et al., 2020). All outcomes and clinical data were extracted from the articles by two investigators (JY Chen and IJ Tsai). The exclusion criteria were as follows: 1) studies including animal or healthy human subjects; 2) studies including pregnant or lactating patients; 3) studies without a controlled group; 4) letters, conference, or case reports; 5) studies that lacked data on mortality and/or did not clearly define ACEi/ARB exposure. Both abstracts and full papers were selected for quality assessment and data syntheses. We contacted the corresponding authors for further data or details, if available.

### Data Analysis and Study Quality Assessment

Two investigators (JY Chen and IJ Tsai) independently reviewed the search results and identified eligible studies. Any resulting discrepancies were resolved by discussion with a third investigator (VC Wu). All relevant data were independently extracted from the included studies by two investigators (JY Chen amd HC Pan) according to a standardized form. Extracted data included study characteristics (leading author, publication year, patient selection, sample size, outcome events, duration of follow-up (years), and the National Clinical Trial number) and participants’ baseline [age (years), gender (%), baseline kidney function (in the form of sCr and/or estimated GFR), comorbidities, and severity of the illness]. When available, odds ratios (OR) and 95% confidence intervals (CIs) from the cohort or case-controlled studies were extracted. Other *a priori* determined parameters were the naive or continuous usage of ACEi/ARB after AKI, different types of ACEi/ARB, duration of follow-up, and the proportions of patients that required intensive care unit care. The baseline characteristics of included studies are illustrated in Table 1.

**TABLE 1:** Baseline characteristics of included studies.

Author	Population	Age (years)	Female (%)	Mean/median eGFR (mL/min/1.73 m^2^)	HTN (%)	DM (%)	CAD (%)	CHF (%)	Stroke (%)	Chronic lung disease (%)	Study design
Brar et al. (2018)	A: 22,193	68.6 ± 16.4	47.2	67.8 ± 27.3	75.9	38.2	11.4	29.2	20.9	NR	Retrospective cohort study
	C: 24,060										
Gayat et al. (2018)	A: 109	67 (55–76)	35.2	52.6	55.6	25.2	NR	11.2	NR	12.3	Prospective study
	C: 502										
Scarton et al. (2019)	A: 45	NR	35.3	NR	21	13.8	15.2	14.7	NR	NR	Prospective cohort study
	C: 303										
Hsu et al. (2020)	A: 1853	62 ± 19	54	NR	38	12	2	NR	1	21	Cohort study
	C: 8,389										
Hines et al. (2020)	A: 72	61.4 ± 14.7	36.2	73.4* (52.4–94.7)	69.9	39.4	30.7	23.8	NR	13.9	Prospective cohort study
	C: 273										
Qiao et al. (2020)	A: 2,674	73.7 ± 12.6	61.6	24.8 ± 4.9	NR	49	44.2	31	20.4	NR	Retrospective study
	C: 1,235										
Bidulka et al. (2020)	A: 3,855	76.6	57.8	54	86.8	46	48.5	33.7	20.9	NR	Population-based cohort
	C: 5,238										

A, ACEi/ARB; C, control; CAD, coronary artery disease; CHF, congestive heart failure; DM, diabetes mellitus; eGFR, estimated glomerular filtration rate (mL/min/1.73 m2); HTN, hypertension; NR, not reported; * median eGFR.

### Quality Assessment

We used the Newcastle-Ottawa Scale to assess the risk of bias of included studies. The following eight domains were evaluated for included observational studies: the representativeness of exposed cohort, selection of nonexposed cohort, ascertainment of exposure, outcome of the interest not present at the start of the study, comparability of cohorts, assessment of outcome, follow-up duration, and adequacy of follow-up of cohorts.

### Data Synthesis and Statistical Analysis

Analyses were performed for exposure to ACEi/ARB after AKI and compared with the control group. Fixed-effect and random-effect (time-variable exposure to ACEi/ARB and follow-up for outcomes) models were used to analyze selected outcomes (all-cause mortality and adverse kidney events) between the two groups. The effect size is expressed as the pooled OR and 95% CIs. We conducted a meta-regression to evaluate possible reasons for differences in ACEi/ARB OR across studies. We also investigated the risk of hyperkalemia related to ACEi/ARB exposure, given that this is an important concern raised by clinicians for the use of these medications after AKI. Funnel plots and Egger’s test were used to examine potential publication bias. Between-trial heterogeneity was determined by using I^2^ tests and values >50% were regarded as considerable heterogeneity. Statistical significance was defined as *p*-values < 0.05, except for the determination of publication bias that employed *p* < 0.10. We used STATA (Version 16, Stata Corp. 2019, College Station, TX, Stata Corp LP) for all statistical analyses.

## Results

### Study Search Outcomes and Included Patients

**FIGURE 1:**
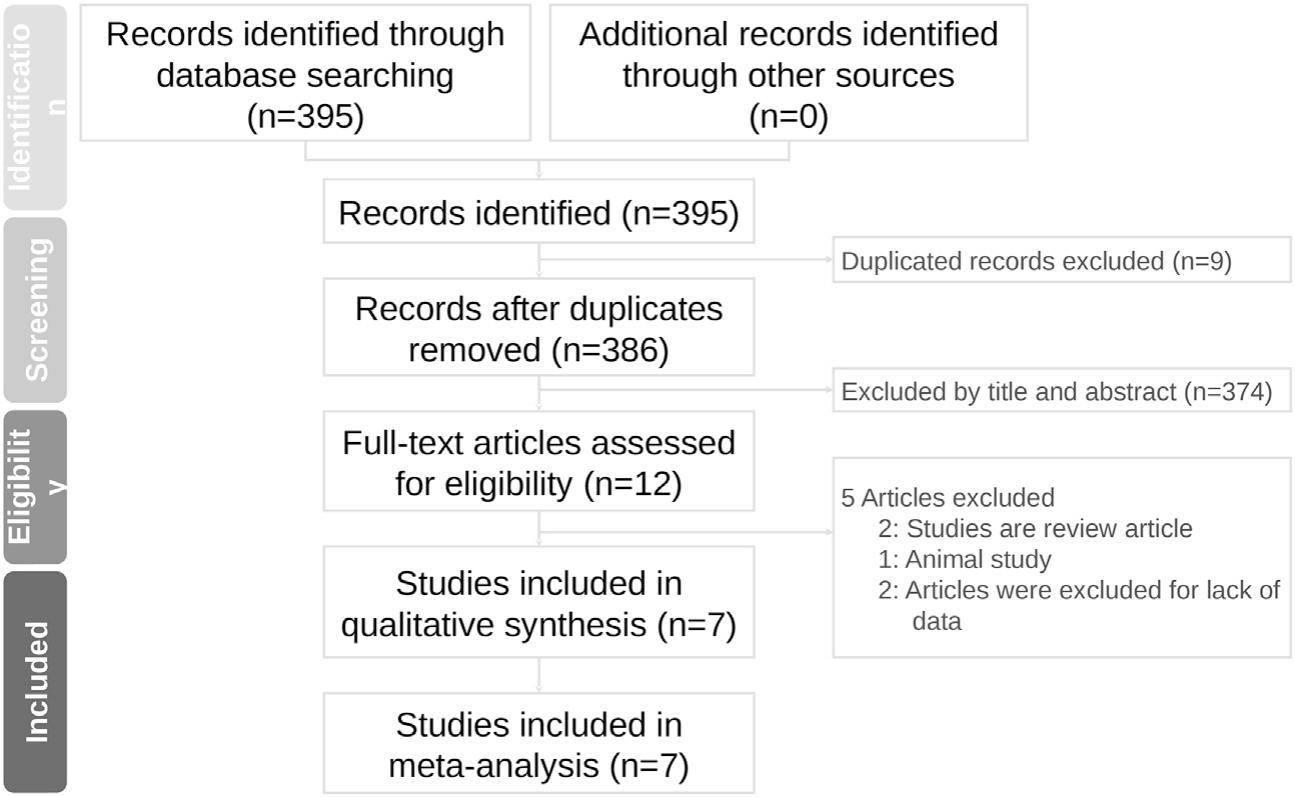
Flowchart of study selection for meta-analysis.

Initially, our search identified 395 articles, of which 363, 27, and five were from PubMed, Embase, and Medline, respectively, but no article was included from Cochrane databases. A total of nine articles were excluded because of duplication; therefore, 386 articles were screened based on their titles and abstracts. Afterward, 12 articles were assessed for full eligibility and five articles were excluded (two were review articles (Dudoignon et al., 2019; Coca, 2020), one was an animal experiment (Cheng et al., 2016), and two lacked critical data (Alpern and Peixoto, 2018; Menez and Parikh, 2020)). Finally, a total of seven articles with complete data were selected for this meta-analysis (Figure 1). The number of patients included in each study ranged from 345 to 46,253, and patient ages ranged from 46.7 to 85 years. All articles compared exposure to ACEi/ARB in the context of AKI with a control group without the exposure. The definition of AKI from the majority of studies was according to KDIGO criteria (an increase in sCr level ≥50% from baseline or ≥0.3 mg/dl within 48 h and/or a need for renal replacement therapy (RRT) during index hospitalization) (Brar et al., 2018; Gayat et al., 2018; Scarton et al., 2019; Hines et al., 2020; Hsu et al., 2020), but Scarton et al. selected the AKI individuals with KDIGO stage three only in their study (Scarton et al., 2019), which included more severe AKI patients than other studies that included milder stages of AKI. Besides, the baseline kidney function was not reported by Scarton et al. Qiao et al. included only patients with decreased eGFR below 30 ml/min/1.73 m^2^ (Qiao et al., 2020). Bidulka et al. defined AKI by International Classification of Diseases-10 (ICD-10) codes (Bidulka et al., 2020).

### Study Characteristics

Two studies were retrospective studies (Brar et al., 2018; Qiao et al., 2020), two were cohort studies (Bidulka et al., 2020; Hsu et al., 2020), and the other three were prospective cohort studies (Gayat et al., 2018; Scarton et al., 2019; Hines et al., 2020). All included studies had an exposure vs. control comparison as described previously. Baseline common characteristics of included comparative studies are shown in Tables 1, 2. The seven included studies were published between 2018 and 2020 and enrolled a total of 70,801 AKI patients. Among these patients, there were 30,801 AKI patients exposed to ACEi or ARB after AKI onset, whereas the other 40,000 AKI patients did not receive any of these medications. Among these AKI patients, there were 18,064 patients who were exposed to ACEi/ARB and another 25,396 patients without exposure available for investigating the outcome of all-cause mortality. Besides, there were 18,035 AKI patients exposed to ACEi/ARB and another 25,425 patients without exposure available for investigating the outcome of recurrent AKI and incident CKD. The follow-up durations in six articles ranged from one to five years (Brar et al., 2018; Gayat et al., 2018; Scarton et al., 2019; Bidulka et al., 2020; Hsu et al., 2020; Qiao et al., 2020), but one article reported a median follow-up of 33 days, which was the date of the first office visit after hospital discharge (Hines et al., 2020). Moreover, the range of mean/median baseline eGFR before AKI was 24.8–73.4 ml/min/1.73 m^2^ (Brar et al., 2018; Gayat et al., 2018; Bidulka et al., 2020; Hines et al., 2020; Qiao et al., 2020). However, three articles did not report baseline kidney function (Scarton et al., 2019; Hsu et al., 2020; Qiao et al., 2020). As to when the ACEi/ARB was prescribed after AKI onset, three articles indicated that it was prescribed after hospital discharge (Brar et al., 2018; Bidulka et al., 2020; Hsu et al., 2020), two other articles mentioned that it was prescribed after discharge from intensive care unit (ICU) (to the nursing floors, but still during the index admission) (Gayat et al., 2018; Scarton et al., 2019), one article stated that it was during the AKI episode (Hines et al., 2020), and in another article, ACEi/ARB exposure was kept continuous even when eGFR was declined below 30 ml/min/1.73 m^2^ (Qiao et al., 2020).

**TABLE 2:** Summary of included comparative studies for outcome evaluation.

Author	Study duration	Follow-up (duration)	AKI definition	The time point with ACEi/ARB given after AKI	Primary outcome	Secondary outcome
Brar et al. (2018)	2013.03.31–2015.03.31	Two years	An increase in sCr level ≥50%, or ≥0.3 mg/dl (from baseline, within 48 h) and/or a need for RRT during index hospitalization	After discharge	All-cause mortality	1. Hospitalization for a renal cause
						2. ESRD
						3. Composite outcome of ESRD
						4. Sustained doubling of sCr levels
Gayat et al. (2018)	2011.08–2013.06	One year	KDIGO criteria or need RRT	At ICU discharge	One-year all-cause mortality	NR
Scarton et al. (2019)	2013.09–2016.01	Two years	KDIGO stage 3	At ICU discharge	60-day mortality	Receipt of RRT
Hsu et al (2020)	2006.01.01–2013.12.31	Two years	A sCr rise ≥0.3 mg/dl within 48 h during the hospitalization or ≥50% above baseline/preadmission sCr	At hospital discharge and throughout follow-up	Recurrent AKI	NR
Hines et al. (2020)	2013.06–2018.04	First clinic visits from discharge	KDIGO criteria	During AKI episode	Persistent AKD (final sCr increase >1.5 times above pre-AKI baseline)	Inability to recover kidney function within 50% of baseline eGFR (AKD stage ≥1)
		33 (18–54) days				
Qiao et al. (2020)	2004.01.01–2018.12.31	Five years	eGFR<30	Six months after the eGFR decrease to less than 30 ml/min/1.73 m^2^	Mortality during the five years	MACE and ESRD
Bidulka et al (2020)	English cohort	Two years	Coding AKI by ICD 10	Within 30 days after discharge because of AKI	Heart failure	AKI, stroke, and death
	2010.01.01–2016.12.31					
	Swedish cohort:2006–2011					

AKD, acute kidney disease; AKI, acute kidney injury; ESKD, end-stage kidney disease; ESRD, end-stage renal disease; ICD, International Classification of Diseases; KDIGO, Kidney Disease: Improving Global Outcomes; MACE, major adverse cardiovascular events; NR, not reported; RRT, renal replacement therapy; sCr, serum creatinine.

Of the included articles, the primary outcome was defined as patient mortality in four articles (Brar et al., 2018; Gayat et al., 2018; Scarton et al., 2019; Qiao et al., 2020) and as recurrent AKI or incident CKD in two articles (Hines et al., 2020; Hsu et al., 2020). Mortality and recurrent AKI were both defined as secondary outcomes in another article (Bidulka et al., 2020).

### Quality of Enrolled Trials

The studies were published in the recent three years (2018–2020) and varied significantly in sample sizes (345–46,253 patients). Besides, the data were retrieved from different sources by the authors, including population databases, health insurance systems, multiple hospitals, and/or ICUs. All participants had an episode of AKI and the authors compared the outcomes according to exposure to ACEi/ARB after AKI onset. The score of the Newcastle-Ottawa Scale Quality Assessment for included studies was 4–9 (Supplementary Table S1).

### Heterogeneity and Publication Bias

The heterogeneity for mortality outcome, according to I^2^ test, was 88.0% and for recurrent AKI or AKD was 97.6%. Publication bias, as assessed using funnel plots (Supplementary Figures S1, S2), was not significant. We used meta-regression to investigate the highly observed heterogeneity.

### ACEi/ARB Exposure and Mortality Outcome

**FIGURE 2:**
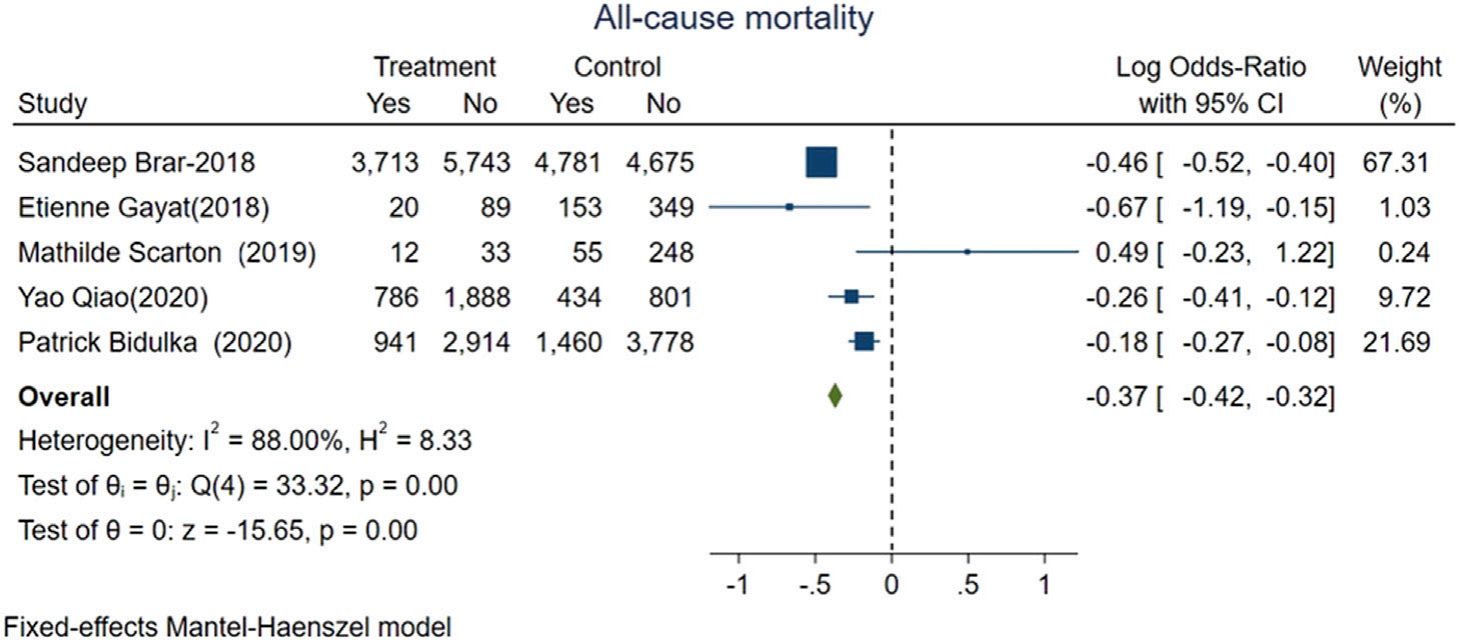
Forest plot showing reduced risk of all-cause mortality comparing ACEi/ARB users vs. nonusers after AKI. Fixed effects of Mantel-Haenszel model.

The main outcome of interest was all-cause mortality, which was evaluated in five included studies representing 43,460 patients and 12,355 deaths. The pooled mortality rate in AKI patients exposed to ACEi/ARB was lower than that in ACEi/ARB nonusers. Specifically, 5,472 patients had mortality in ACEi/ARB group with total 18,064 patients vs. 6,883 patients had mortality in the control group without ACEi/ARB exposure with total 25,396 patients. The pooled logOR were significant for the reduced risk of all-cause mortality with ACEi/ARB exposure after AKI onset [fixed-effect logOR −0.37, 95% CI: −0.42–−0.32, *p* < 0.01 (Figure 2) and random-effect logOR −0.29, 95% CI: −0.48–−0.11, *p* < 0.01(Supplemental Figure 3)]. High heterogeneity was found among included studies (fixed-effect model, I^2^ value of 88.0%, Figure 2; random-effect model, I^2^ value of 86.7%, Supplementary Figure S3).

### ACEi/ARB Exposure and Adverse Kidney Events

**FIGURE 3:**
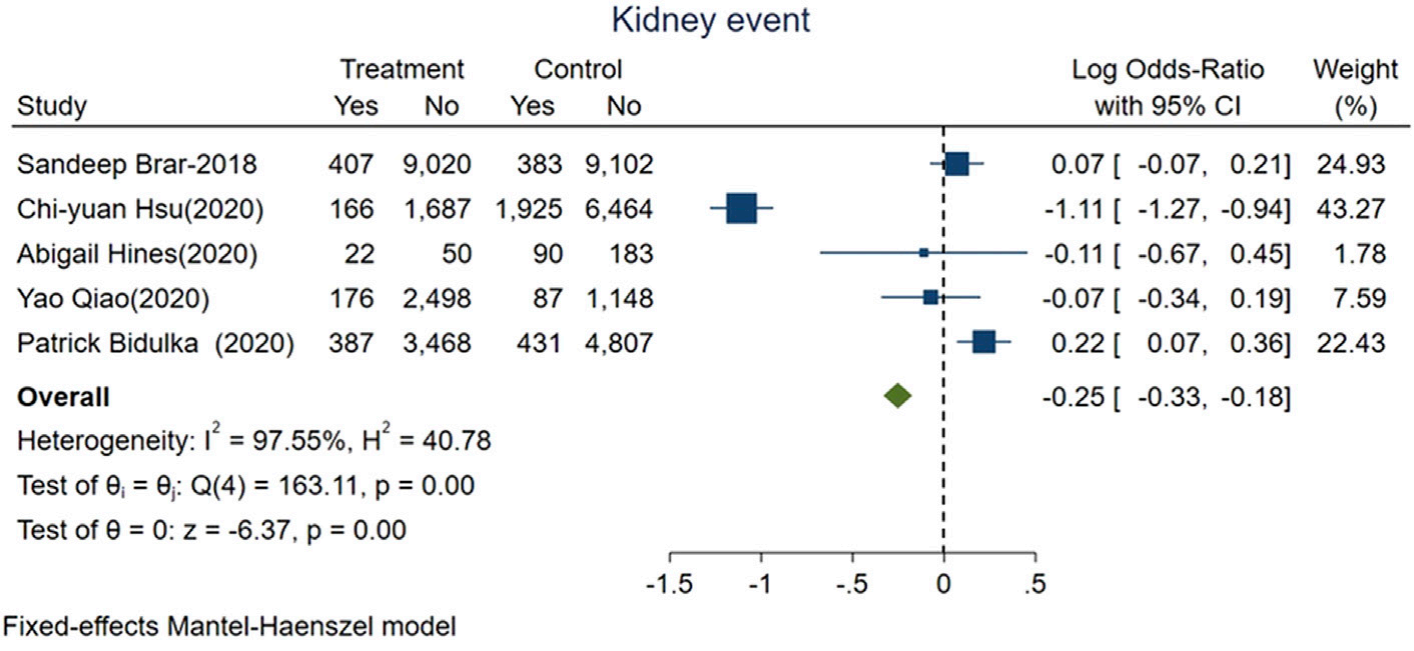
Forest plot showing reduced risk of adverse kidney events comparing ACEi/ARB users vs. nonusers after AKI. Fixed effects of Mantel-Haenszel model.

Our secondary outcome of interest was recurrent AKI or incident CKD or ESKD which were based on five studies representing 43,460 patients, with 4,074 patients who had these adverse kidney events. The pooled events rate was 6.4% in the group of AKI patients who were given ACEi/ARB (1,158 of 18,035) vs. 11.5% (2,916 of 25,425) for those without exposure. The pooled logOR were significant for the reduced risks of adverse kidney events with ACEi/ARB exposure after AKI onset [fixed-effect logOR −0.25, 95% CI: −0.33–−0.18, *p* < 0.01 (Figure 3) and random-effect logOR −0.2, 95% CI: −0.69–0.28, *p* = 0.4 (Supplementary Figure S4)]. High heterogeneity was found among included studies (fixed-effect model, I^2^ value of 97.55%, Figure 3; random-effect model, I^2^ value of 96.70%, Supplementary Figure S4).

### Risk of Hyperkalemia

**FIGURE 4:**
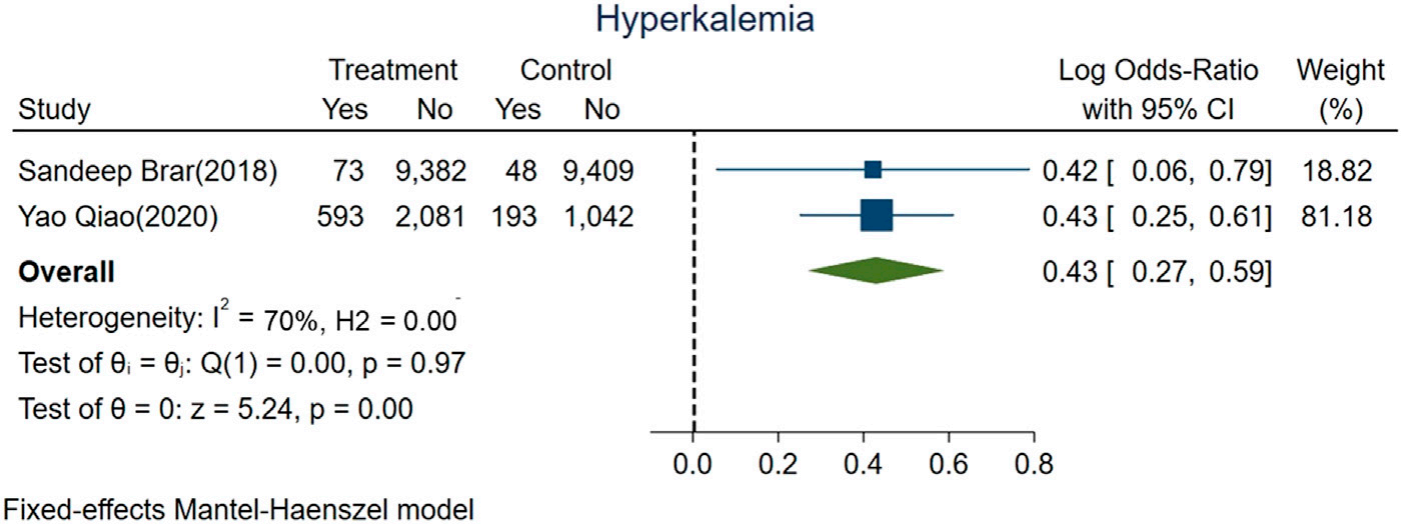
Forest plot showing higher risk hyperkalemia between ACEi/ARB users vs. nonusers after AKI.

We evaluated risk of hyperkalemia based on all included studies representing 22,821 patients; 907 of whom experienced hyperkalemia. The pooled risk of hyperkalemia was 5.5% (666 of 12,129) for ACEi/ARBs users vs. 2.3% (241 of 10,692) for nonusers (logOR 0.43, 95% CI: 0.27–0.59, *p* < 0.01; Figure 4). High heterogeneity was found among studies (fixed-effect model, I^2^ value of 70%, Figure 4).

We also investigate mortality and adverse kidney events among subgroups of AKI patients with or without exposure to ACEi/ARB before AKI happened.

**FIGURE 5:**
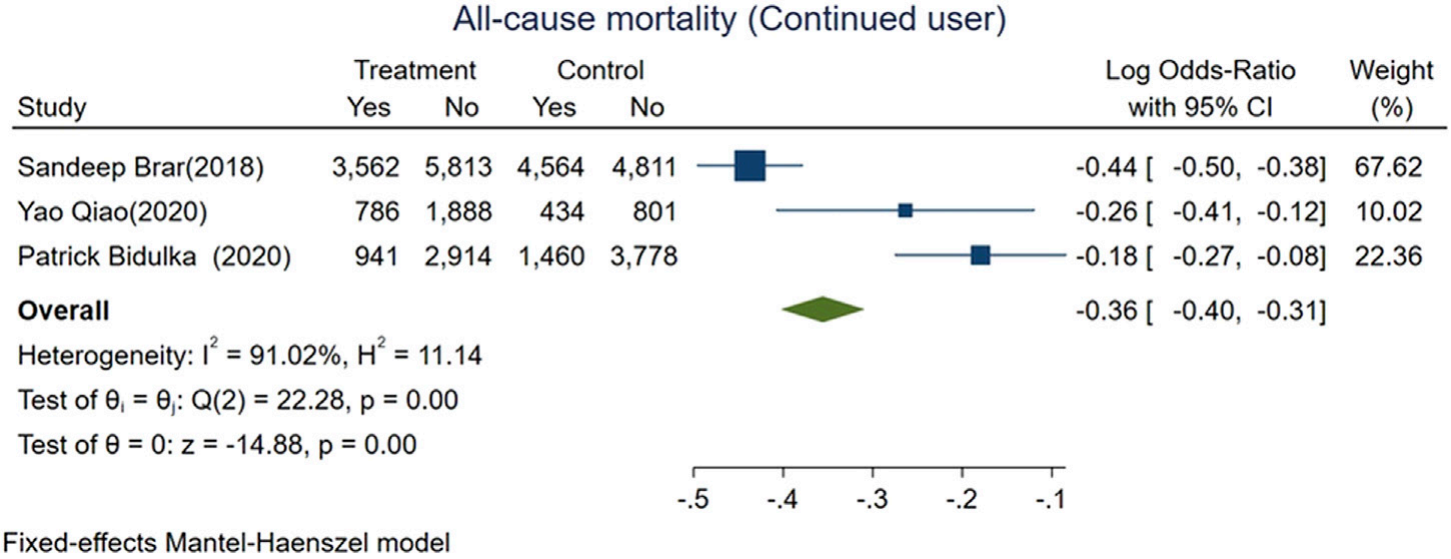
Forest plot for all-cause mortality comparing continued ACEi/ARB use vs. *de novo* use in those exposed to ACEi/ARB after AKI.

**FIGURE 6:**
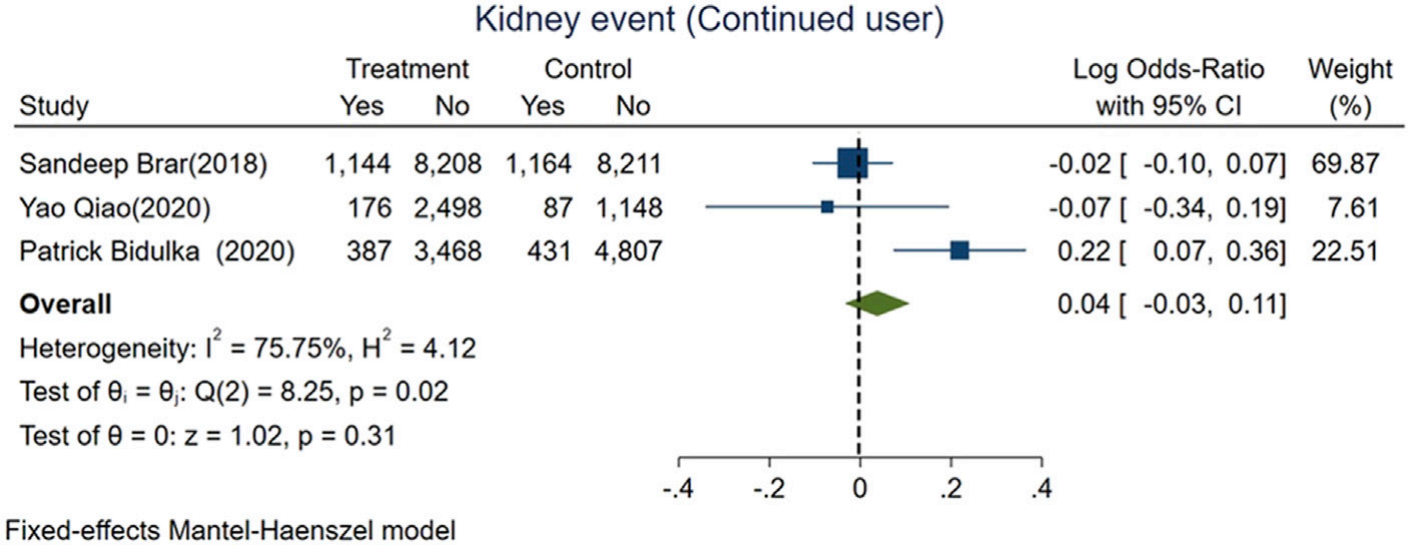
Forest plot for adverse kidney events comparing continued ACEi/ARB use vs. *de novo* use in those exposed to ACEi/ARB after AKI.

We divided AKI patients exposed to ACEi/ARB into two groups: those who had continued usage (patients who were taking the medication before the index AKI episode and were continued on it after the AKI event) and patients who were naive to ACEi/ARB. The mortality risk was lower for those with continued exposure than those who were exposed for the first time after AKI (logOR −0.36, 95% CI: −0.4–−0.31, *p* < 0.01; Figure 5). However, adverse kidney events were similar among both groups (log OR 0.04, 95% CI: −0.03–0.11, *p* = 0.31; Figure 6).

### Meta-Regression Analysis

Increased percentages of congestive heart failure (CHF) and diabetes were associated with attenuated logOR for all-cause mortality between exposed and control groups (Z = 3.31; *p* = 0.001, Supplementary Figure S5A; Z = 3.69; *p* < 0.001, Supplementary Figure S5B, respectively). Increased percentages of diabetes and older age were associated with an attenuated logOR for adverse kidney events between exposed and control groups (Z = 12.11; *p* < 0.001, Supplementary Figure S6A; Z = 10.58; *p* < 0.001, Supplementary Figure S6B, respectively).

### Sensitivity Analysis

We further investigated the possible effect of modification of comorbidities on all-cause mortality using hazard ratio. The subgroup analysis with a hazard ratio for all-cause mortality was conducted for three articles (Brar et al., 2018; Gayat et al., 2018; Qiao et al., 2020). The all-cause mortality rate of ACEi/ARB users after AKI was lower than that of nonusers (log OR:0.80, 95% CI: [0.69–0.91], *p* < 0.01; Supplementary Figure S7).

### Assessment of Evidence Quality and Summary of Findings

Evidence quality assessment was performed using the GRADE system. We evaluated the primary outcome and secondary outcomes and present a summary of these findings in the supplementary file.

## Discussion

In this systematic review of seven studies comprising 70,801 patients that had AKI, the pooled mortality rate and adverse kidney events after AKI were 28.4 and 9.4%, respectively, and 6.4% were exposed to ACEi/ARB after AKI. We found that ACEi/ARB users had a lower risk of all-cause mortality and recurrent/worsening adverse kidney events compared with ACEi/ARB nonusers. The results also revealed a higher risk of hyperkalemia when exposed to these medications after AKI, implicating caution needed in monitoring for hyperkalemia when these medications are initiated/restarted after AKI. Interestingly, we found that, among patients exposed to ACEi/ARB after AKI, those with continued exposure (taking these medications prior to the AKI event) had a lower risk of death than those who were exposed to these medications for the first time after AKI. These results should be interpreted with caution as current literature is highly heterogeneous, although funnel meta-regression analysis showed only limited publication bias. Thus far, to our knowledge, this is the most comprehensive and unprecedented systematic review that addresses these important questions that directly affect post-AKI care.

After AKI episodes, it has been proposed that the transition period between AKI and possibly CKD, referred now to as acute kidney disease (AKD; 7–90 days after AKI), may be an opportunity to intervene, and the patients’ future progression could be altered. Emerging data suggest that strategies for better blood pressure control, which could be facilitated *via* the initiation/reinitiation of ACEi/ARB (Cheng et al., 2016; Brar et al., 2018), may prevent the progression of kidney disease and its related morbidity and mortality. However, these observations have not been rigorously tested in interventional studies. Further, there were no effective pharmacological interventions to mitigate the incidence or progression of AKD or AKI-to-CKD progression. With the results of this study, we underpin the potential benefits of ACEi/ARB in lowering the risk of mortality and adverse kidney events after AKI, but we also need close monitoring due to the observed increased risk of hyperkalemia among ACEi/ARB users (Brar et al., 2018).

### ACEi/ARB Effects on All-Cause Mortality and Adverse Kidney Events

AKI survivors are at higher risk of developing hypertension, stroke, and long-term cardiovascular events (Chawla et al., 2014; Wu et al., 2014; Omotoso et al., 2016; Ozrazgat-Baslanti et al., 2016). Therefore, the use of antihypertensive medication(s) for post-AKI/AKD care, specifically ACEi/ARB, becomes an important aspect for best clinical practices to scrutinize. Timely use of ACEi/ARB after AKI may also prevent heart failure hospitalization, a common cause of morbidity among these patients, due to its effect on afterload reduction (Silver et al., 2017; Go et al., 2018). Overall, our data showed that the use of ACEi/ARB should be timely favored after an episode of AKI because it may improve clinical outcomes in these patients. With our findings, careful clinical reevaluation and timely initiation/reinitiation of ACEi/ARB among the post-AKI/AKD patients are to be encouraged in order to improve the prognosis of AKI patients (Wu et al., 2020).

ACEi/ARB may reduce tubular damage during AKI insults by keeping peritubular capillary perfusion *via* efferent arteriolar vasodilation and therefore increasing the renal medullary plasma flow by diminishing the filtration fraction (Li et al., 2012). It has been shown that angiotensin II blockade reduces tubular ischemia and development of acute tubular necrosis or injury (Norman et al., 2003). In addition, the use of ACEi/ARB is recommended for diabetic nephropathy to limit the progression of kidney disease (Whelton et al., 2018). Moreover, ACEi/ARB also reduces mortality due to cardiovascular disease, including myocardial infarction and CHF (Ambrosioni et al., 1995). Overall, evidence supports the use of ACEi/ARB due to kidney and cardiovascular protection and decreased all-cause mortality. The results from our current meta-analysis were in accordance with the prior reports and gave strong support for the timely use after AKI. AKI has a significant impact on the functions or injury/repair pathways on remote organs (Dépret et al., 2017), and activation of RAAS could result in profibrotic pathways directly affecting vital organs. On the basis of such evidence, we speculated that the use of ACEi/ARB may prevent maladaptive repair and improve organ function after AKI (Zhong et al., 2010; Simões and Teixeira, 2016).

We also provide evidence that CHF and diabetes may significantly modify the association between exposure to ACEi/ARB and mortality. Specifically, the observed survival benefit of ACEi/ARB after AKI was more obvious in patients without prevalent CHF and diabetes. Similarly, the observed benefit of ACEi/ARB on adverse kidney events after AKI was more obvious in younger patients and those without diabetes. The concerns regarding altered renal hemodynamics and autoregulation by ACEi/ARB effect on efferent arteriole vasodilation have supported the common clinical practice of stopping or avoiding the use of these medications in the context of AKI (namely, sick-day rule) (Whiting et al., 2017). This finding was supported by the fact that RAAS inhibitors could improve kidney oxygenation in patients with CKD, especially of the medulla (Lin et al., 2019). In patients with CHF, it has been demonstrated that the use of ACEi/ARB reduces mortality and CHF-related rehospitalization, as well as improving the quality of life (Emdin et al., 2015). However, the use of ACEi/ARB lowers blood pressure and along with preexisting CHF-related low-flow status could increase the risk of kidney hypoperfusion and worsening kidney function. Nonetheless, acute declines in GFR from ACEi/ARB are known to be related to transient kidney perfusion but not tubular injury/damage (intrinsic AKI) (Gayat et al., 2018). The balance between risks and benefits of ACEi/ARB in hospitalized patients (even in those with AKI) should be made, allowing for “permissive AKI” or “permissive hypercreatinemia” in some cases to favor long-term cardiovascular, kidney, and mortality outcomes (Parikh and Coca, 2019). Additionally, reduction of renal medullary circulation was associated with elevation of superoxide or decrease of nitric oxide (NO), which interacts with angiotensin II (Mori et al., 2006). Gupta et al. demonstrated that administration of an ACEi, captopril, offers protection against the development of contrast-induced nephrotoxicity (Gupta et al., 1999). Such evidence showed that the possible role of medullary ischemia mediated by RAAS (Gupta et al., 1999) could be alleviated by ACEi/ARB.

We emphasized the importance of controlling underlying diseases, such as CHF and diabetes, which still had a significant impact on mortality even if ACEi/ARB was prescribed. Similarly, the finding of “the use of ACEi/ARB had more survival benefits on the younger patients with fewer comorbidities or patients without diabetes” also pointed to more cautious usage of ACEi/ARB in comorbidities, especially those with CHF or diabetes.

### Strengths and Limitations

To the best of our knowledge, this is the first systematic review and meta-analysis evaluating whether various ACEi/ARB exposure after AKI is associated with better outcomes in terms of mortality and adverse kidney events. Our findings represent the best current evidence supporting the potential benefit of ACEi/ARB after AKI for the amelioration of relevant clinical outcomes such as all-cause mortality, recurrent AKI/AKD, or CKD/ESKD progression. We also identified critical effect modifiers such as diabetes, CHF, and age that should be considered when evaluating these outcomes. We also cautioned of the higher risk of hyperkalemia episodes in patients exposed to ACEi/ARB after AKI. We carefully evaluated current evidence, determined inclusion criteria, and adapted the GRADE approach to rate the certainty of evidence (Guyatt et al., 2011). Finally, we also identified and addressed a detailed body of published work from China, especially evidence in the Chinese language mostly emerged from the effect of RAAS inhibitors on outcomes in CKD patients (Hou et al., 2006).

Some limitations of our study are notable. First, there were different illness severities for each study, such as those from ICU patients (Gayat et al., 2018; Scarton et al., 2019), those from population-based databases (Brar et al., 2018; Hsu et al., 2020; Qiao et al., 2020), or those from hospitalized patients without mention of location (Hines et al., 2020). Second, the baseline kidney function and severities of kidney injury were heterogeneous and there were three studies (Scarton et al., 2019; Hsu et al., 2020; Qiao et al., 2020) without reporting of the baseline sCr levels. These factors might account for some of the residual statistical heterogeneity seen for some outcomes. However, one should note that I^2^ is commonly inflated in meta-analyses of observational data (Iorio et al., 2015). Third, the different time points of prescribing ACEi/ARB after the index AKI episode may have unfathomed exposure for outcome evaluation. Although it was not possible to conclusively ascertain sources of heterogeneity, we conducted subgroup analysis and meta-regression for potential publication bias. Fourth, prescribing indications among those with ACEi/ARB exposure were not elucidated and that could be a source of residual unmeasured confounding. Further, the use of ACEi/ARB may represent a subgroup of patients with relatively stable hemodynamics and differential risk of post-AKI outcomes. To decrease the confounding nature by prescribing indications, we analyzed the adjusted OR together with variable subgroup analysis, and the results showed consistency with our main findings. Fifth, this study is dampened by the retrospective nature in five out of seven included studies and the inherent limitations of such designs. However, caution should be admonished regarding these associations because of potential unmeasured residual confounding given the observational designs of the studies and the small numbers of patients in the secondary outcome analysis, especially the different definitions of adverse kidney events. Sixth, there was insufficient information to evaluate the prognostic impact of different definitions of hyperkalemia. Seventh, we could not get enough information on the medications that would interfere with the effect of the RAAS, e.g., beta-blocker, angiotensin receptor-neprilysin inhibitor, and SGLT2. Further studies are necessary to examine the effect of coadministration of these medications and ACEi/ARB. Eighth, the effects of another type of medication that would also interact with the RAAS—mineralocorticoid receptor antagonist (MRA)—on the same outcomes were not specifically measured in this study, partially because of the seventh limitation mentioned previously that not all studies recorded clearly the use of other concomitant medications to control the blood pressure, partially because of the fact that even there were data regarding the protective effect of MRA on diabetic CKD patients (Currie et al., 2016; Alexandrou et al., 2019; Bakris et al., 2020), the impact of MRA on “AKI or AKD” was not established comprehensively. Lin et al. (2019) reported that previous MRA users with AKD had a higher risk of hyperkalemia compared with nonusers but there was similar risk for incident major adverse cardiovascular events (MACE) and death between two groups. We need more studies to investigate the outcome of MRA for AKI or AKD patients.

## Conclusion

Our comprehensive systematic review and meta-analysis provided the best available information on treatment with ACEi/ARB after AKI and concluded that exposure to these medications after AKI may decrease the risk of all-cause mortality, recurrent AKI/AKD, or CKD/ESKD progression in this susceptible population. We also pointed out the concern of a higher risk of hyperkalemia with ACEi/ARB use after AKI, which requires periodical electrolytes monitoring among these patients. Despite the fact that interventional studies testing timely exposure to ACEi/ARB in the context of AKI are direly needed, this systematic appraisal of currently best available evidence could be considered informative interim guidance. Further studies should be conducted to confirm these results.

## Data Availability

The original contributions presented in the study are included in the article/Supplementary Material, and further inquiries can be directed to the corresponding author.
