# Supplementary material for: The Impact of Angiotensin-Converting Enzyme Inhibitors or Angiotensin II Receptor Blockers on Clinical Outcomes of Acute Kidney Disease Patients: A Systematic Review and Meta-Analysis
Source: Front Pharmacol. 2021 Jul 20;12:665250. doi: 10.3389/fphar.2021.665250 (PMC8329451; doi:10.3389/fphar.2021.665250)
Supplement: Supplementary file 1 [file DataSheet1.docx]

**Supplementary appendix**

This supplementary appendix provides:

1. Search equation via PubMed, EMBASE, MEDLINE, and [Cochrane](http://www.cochranelibrary.com/) library
2. Quality assessment of the included studies.
3. PRISMA checklist.
4. Subgroup analysis of the forest plot of odds ratio (OR) and 95% confidence interval (CI).
5. Summary of contextual factor data.
6. PROSPERO protocol registration.
7. Quality assessment the GRADE results.
8. Flow chart showing Search strategy for studies in China.
9. **Search equation via PubMed, EMBASE, MEDLINE, and** [**Cochrane**](http://www.cochranelibrary.com/) **library**

**Appendix.**

Search strategies for the different databases ran on July 21, 2020.

**PubMed Search Query**

#1. (((((((("angiotensin converting enzyme inhibitors"[Pharmacological Action] OR "angiotensin-converting enzyme inhibitors"[MeSH Terms]) OR (("angiotensin converting"[All Fields] AND "enzyme"[All Fields]) AND "inhibitors"[All Fields])) OR "angiotensin converting enzyme inhibitors"[All Fields]) OR ((("angiotensin"[All Fields] AND "converting"[All Fields]) AND "enzyme"[All Fields]) AND "inhibitors"[All Fields])) OR "angiotensin converting enzyme inhibitors"[All Fields]) OR ((((("receptors, angiotensin"[MeSH Terms] OR ("receptors"[All Fields] AND "angiotensin"[All Fields])) OR "angiotensin receptors"[All Fields]) OR ("angiotensin"[All Fields] AND "receptor"[All Fields])) OR "angiotensin receptor"[All Fields]) AND (("blocker"[All Fields] OR "blocker s"[All Fields]) OR "blockers"[All Fields]))) OR ((((("renin-angiotensin system"[MeSH Terms] OR ("renin angiotensin"[All Fields] AND "system"[All Fields])) OR "renin angiotensin system"[All Fields]) OR ((("renin"[All Fields] AND "angiotensin"[All Fields]) AND "aldosterone"[All Fields]) AND "system"[All Fields])) OR "renin angiotensin aldosterone system"[All Fields]) AND (("blocker"[All Fields] OR "blocker s"[All Fields]) OR "blockers"[All Fields]))) OR ((((("renin-angiotensin system"[MeSH Terms] OR ("renin angiotensin"[All Fields] AND "system"[All Fields])) OR "renin angiotensin system"[All Fields]) OR (("renin"[All Fields] AND "angiotensin"[All Fields]) AND "system"[All Fields])) OR "renin angiotensin system"[All Fields]) AND ((("blockade"[All Fields] OR "blockaded"[All Fields]) OR "blockades"[All Fields]) OR "blockading"[All Fields]))) AND ((((("acute kidney injury"[MeSH Terms] OR (("acute"[All Fields] AND "kidney"[All Fields]) AND "injury"[All Fields])) OR "acute kidney injury"[All Fields]) OR (((("acute kidney injury"[MeSH Terms] OR (("acute"[All Fields] AND "kidney"[All Fields]) AND "injury"[All Fields])) OR "acute kidney injury"[All Fields]) OR (("acute"[All Fields] AND "renal"[All Fields]) AND "failure"[All Fields])) OR "acute renal failure"[All Fields])) OR ((("mortality"[MeSH Terms] OR "mortality"[All Fields]) OR "mortalities"[All Fields]) OR "mortality"[MeSH Subheading])) OR (((((("advance"[All Fields] OR "advanced"[All Fields]) OR "advancement"[All Fields]) OR "advancements"[All Fields]) OR "advances"[All Fields]) OR "advancing"[All Fields]) AND (((("kidney diseases"[MeSH Terms] OR ("kidney"[All Fields] AND "diseases"[All Fields])) OR "kidney diseases"[All Fields]) OR ("kidney"[All Fields] AND "disease"[All Fields])) OR "kidney disease"[All Fields]))) (10628)

**EMBASE**

No. Query Results (579)

(((angiotensin AND converting AND enzyme AND inhibitors OR angiotensin) AND receptor AND blocker OR renin) AND angiotensin AND aldosterone AND system AND blockers OR 'renin angiotensin') AND system AND blockade AND ((acute AND kidney AND injury OR acute) AND renal AND failure OR mortality OR advanced) AND kidney AND disease

**Medline (EBSCO) Search Query**

#1( (angiotensin converting enzyme inhibitors OR Angiotensin Receptor Blocker OR Renin angiotensin aldosterone system blockers OR Renin-Angiotensin System Blockade) ) AND ( (acute kidney injury OR acute renal failure OR mortality OR Advanced kidney disease) ) (72961)

Expanders - Apply related words; Also search within the full text of the articles; Apply equivalent subjects
Narrow by Language: - English
Search modes - Find all my search terms

**Cochrane Library**

#1 (angiotensin converting enzyme inhibitors OR Angiotensin Receptor Blocker OR Renin angiotensin aldosterone system blockers OR Renin-Angiotensin System Blockade) in All Text AND (acute kidney injury OR acute renal failure OR Advanced kidney disease) in All Text OR mortality in All Text (4309)

1. **Quality assessment of the included studies**

**Supplemental Table 1. Newcastle-Ottawa Scale Quality Assessment of included studies**

|  | **Selection** | | | | **Comparability** | | **Exposure** | | | | |  |
| --- | --- | --- | --- | --- | --- | --- | --- | --- | --- | --- | --- | --- |
| First author / Year | **Representativeness of the exposed cohort** | **Selection of the non exposed cohort** | **Ascertainment of exposure** | **Demonstration that outcome of interest was not present at start of study** | **Comparability of cohorts on the basis of the design or analysis** | | **Assessment of outcome** | **Was follow-up long enough for outcomes to occur** | | | **Adequacy of follow up of cohorts** | **Total** |
| Sandeep Brar / 2018 | * | * | * | - | | * | * | | * | * | | 7 |
| Etienne Gayat/ 2018 | * | * | * | * | | * | - | | * | - | | 6 |
| Mathilde Scarton1/2019 | - | * | * | - | | * | - | | * | - | | 4 |
| Chi-yuan Hsu/2020 | * | * | * | * | | * | * | | * | * | | 8 |
| Abigail Hines/2020 | * | * | * | * | | ** | * | | * | * | | 9 |
| Yao Qiao/2020 | * | * | * | - | | * | * | | * | * | | 7 |
| Patrick Bidulka/2020 | * | * | * | * | | * | * | | * | - | | 8 |

1. **PRISMA checklist**

**
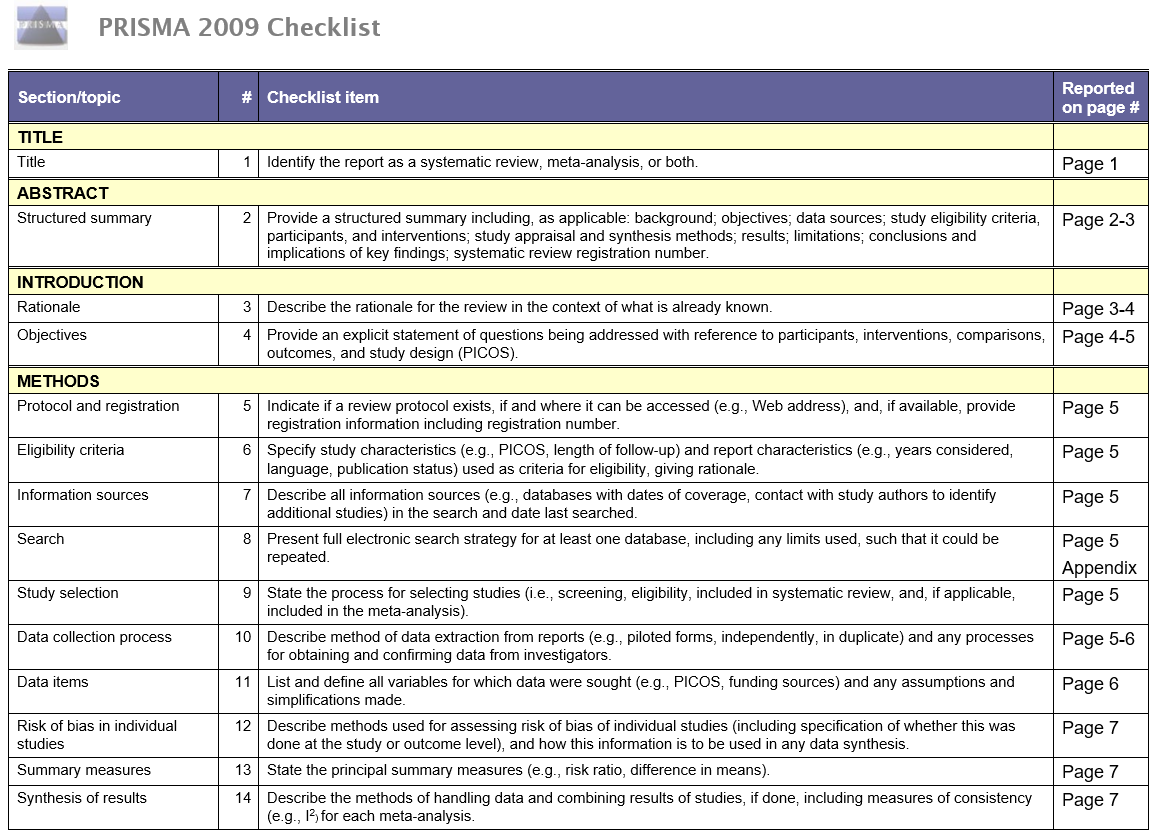
**

**
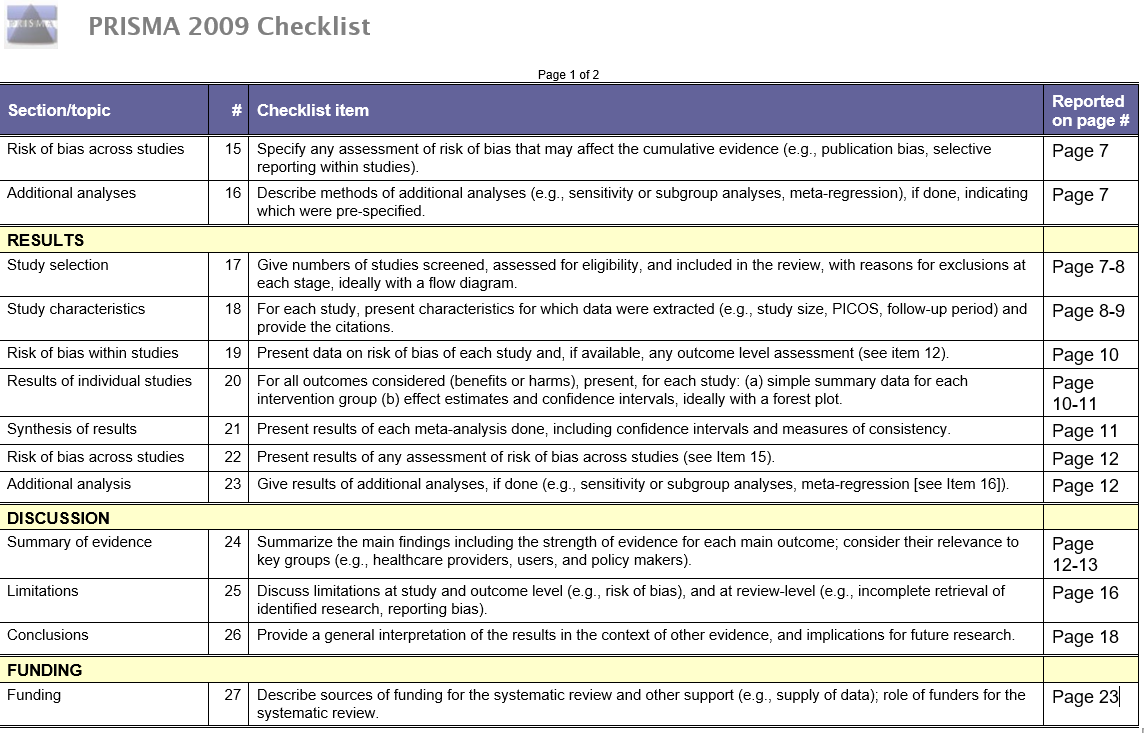
**

1. **Subgroup analysis of the forest plot of odds ratio (OR) and 95% confidence interval (CI)**

**
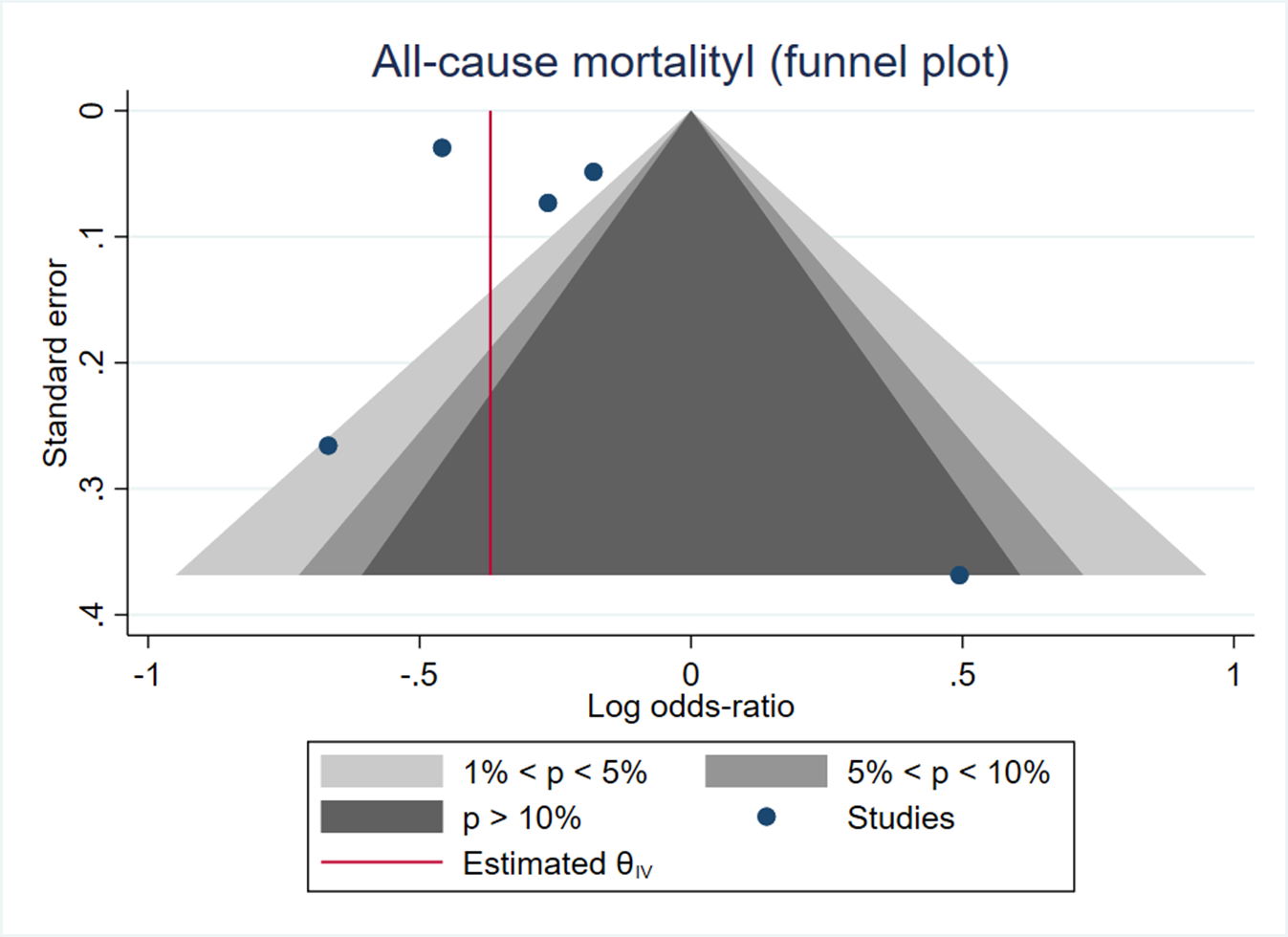
**

**Supplemental Figure 1.** The funnel plot showing the visual check for publication bias of the effect of ACEi/ARB use after AKI on the risk of all-cause mortality by pooling the adjusted odds ratios.


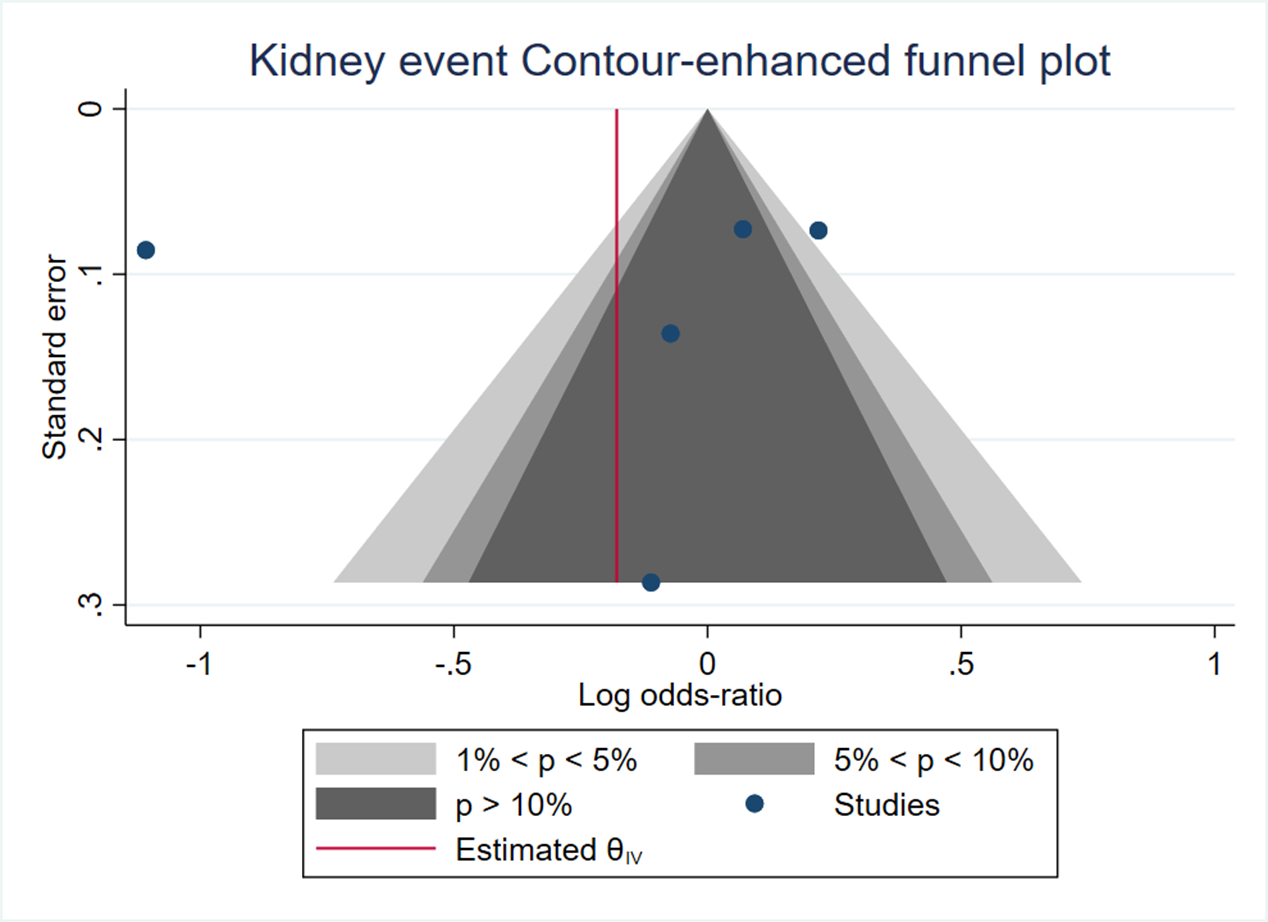


**Supplemental Figure 2.** The funnel plot showing the visual check for publication bias of the effect of ACEi/ARB after AKI use on the risk of adverse kidney events by pooling the adjusted odds ratios.

**
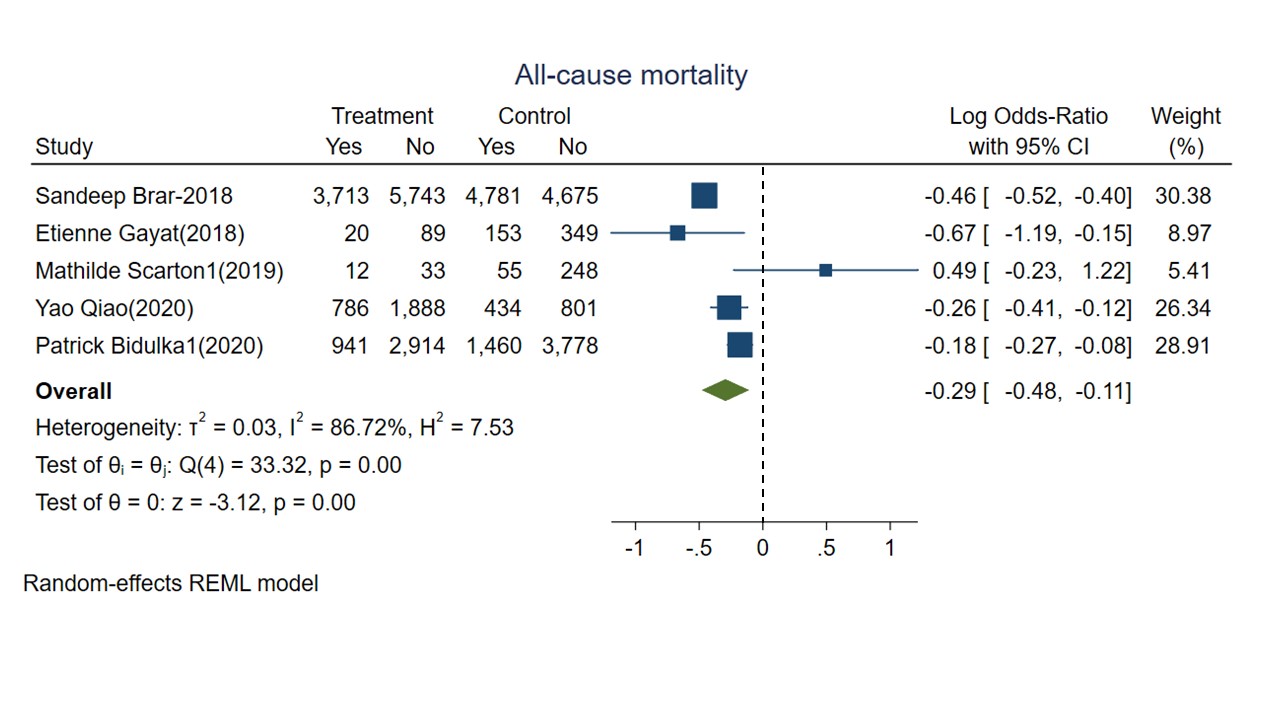
**

**Supplement Figure 3.** Forest plot for all-cause mortality comparing ACEi/ARB user versus nonuser after AKI. Random-effects of REML model


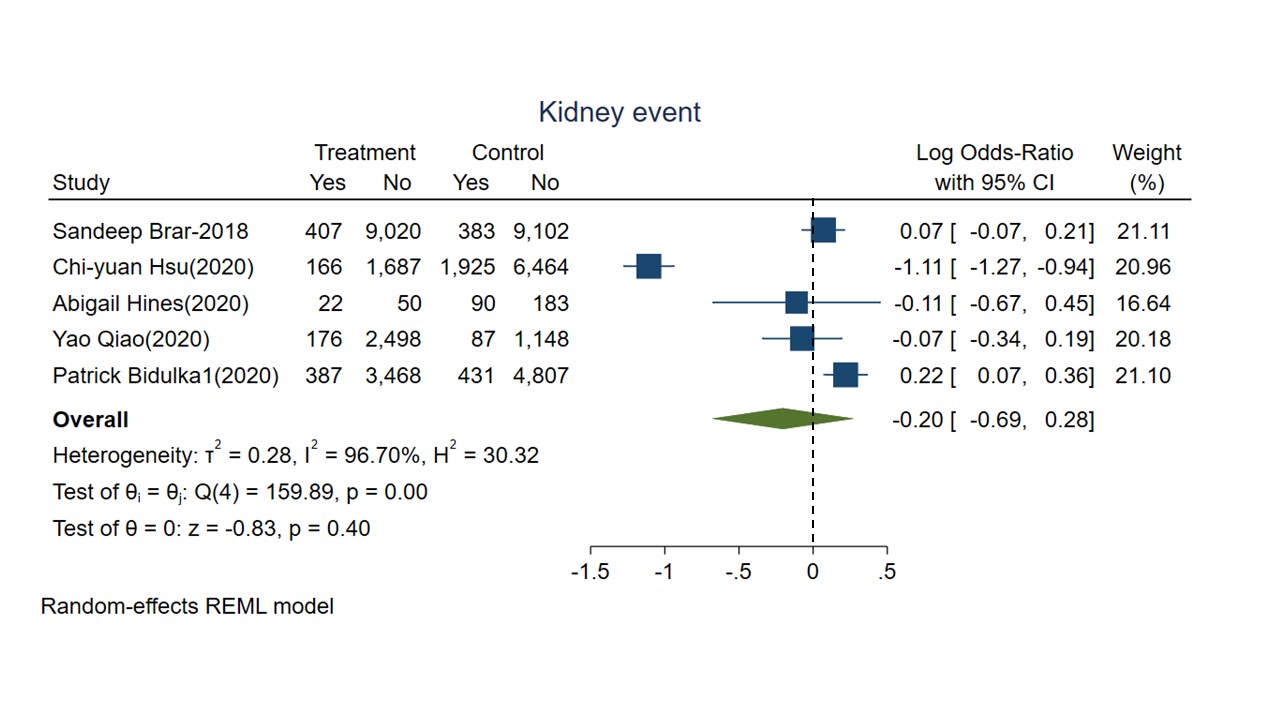


**Supplemental Figure 4.** Forest plot for adverse kidney events comparing ACEi/ARB user versus nonuser after AKI. Random-effects of REML model

**Supplemental Figure 5A**

**
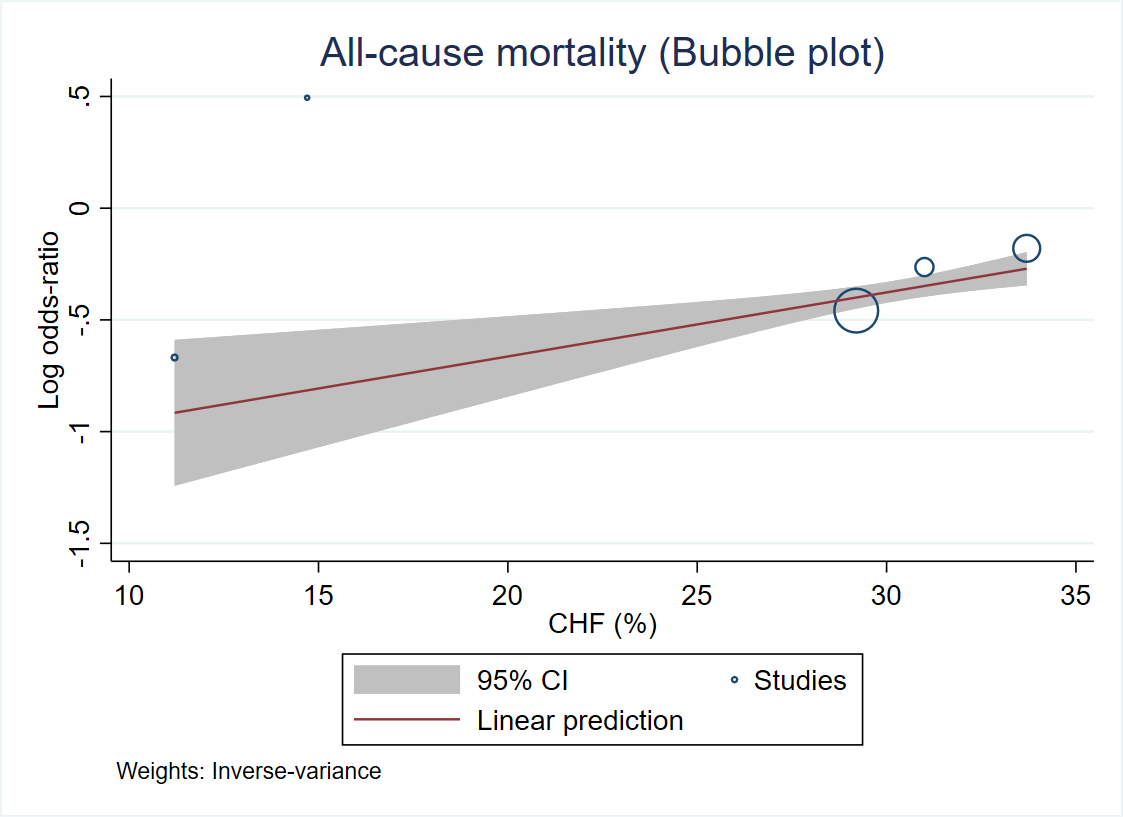
**

**Supplemental Figure 5B**

**
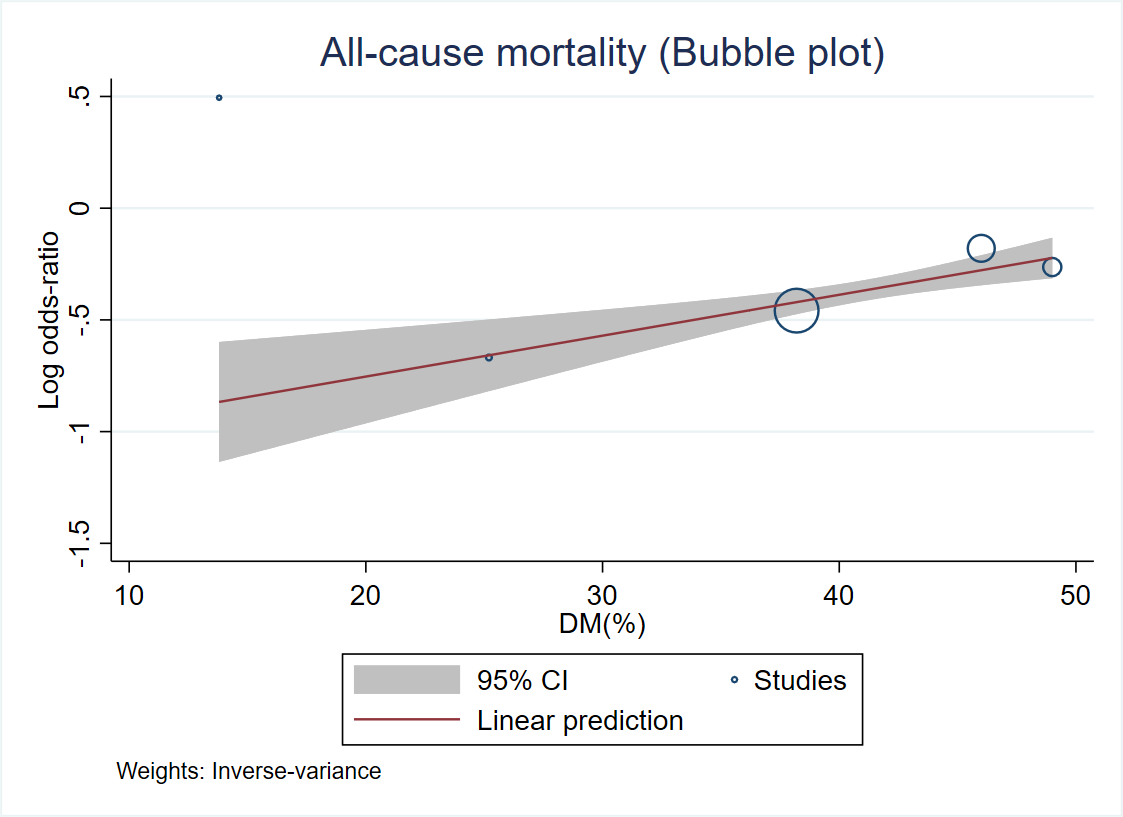
**

**Supplement Figure 5**

The meta-regression bubble plot showing the effect modification of frequency of (A) Congestive Heart Failure and (B) Diabetes in included studies and the risk of all-cause mortality according to ACEi/ARB exposure after AKI.

**Supplement Figure 6A**

**
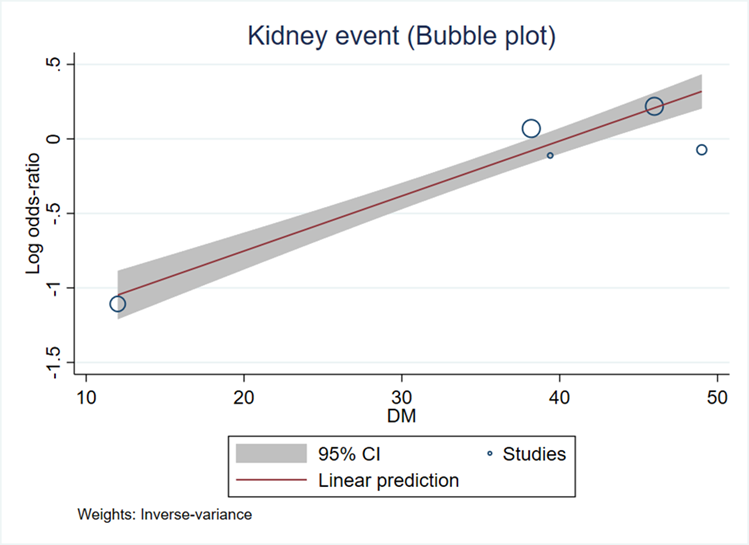
**

**Supplement Figure 6B**

**
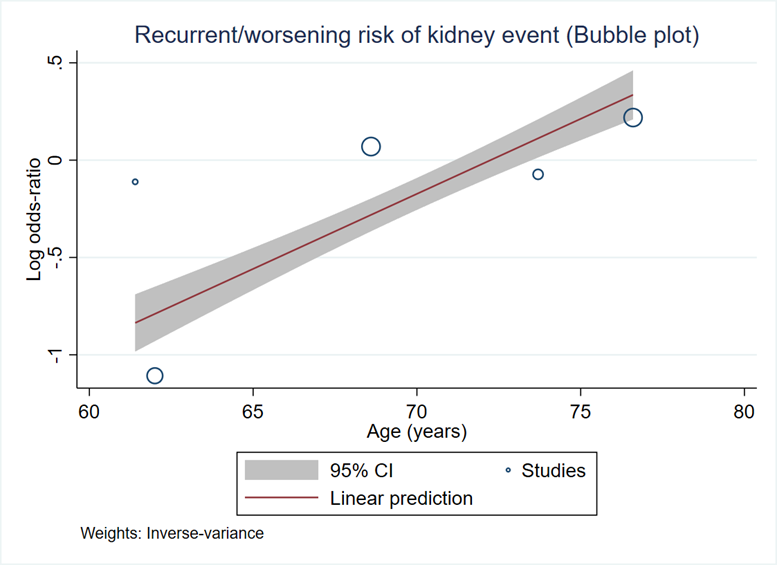
**

**Supplement Figure 6**

The meta-regression bubble plot showing the effect modification of frequency of (A) Diabetes and impact of (B) Age in included studies and adverse kidney events according to ACEi/ARB exposure after **AKI**.


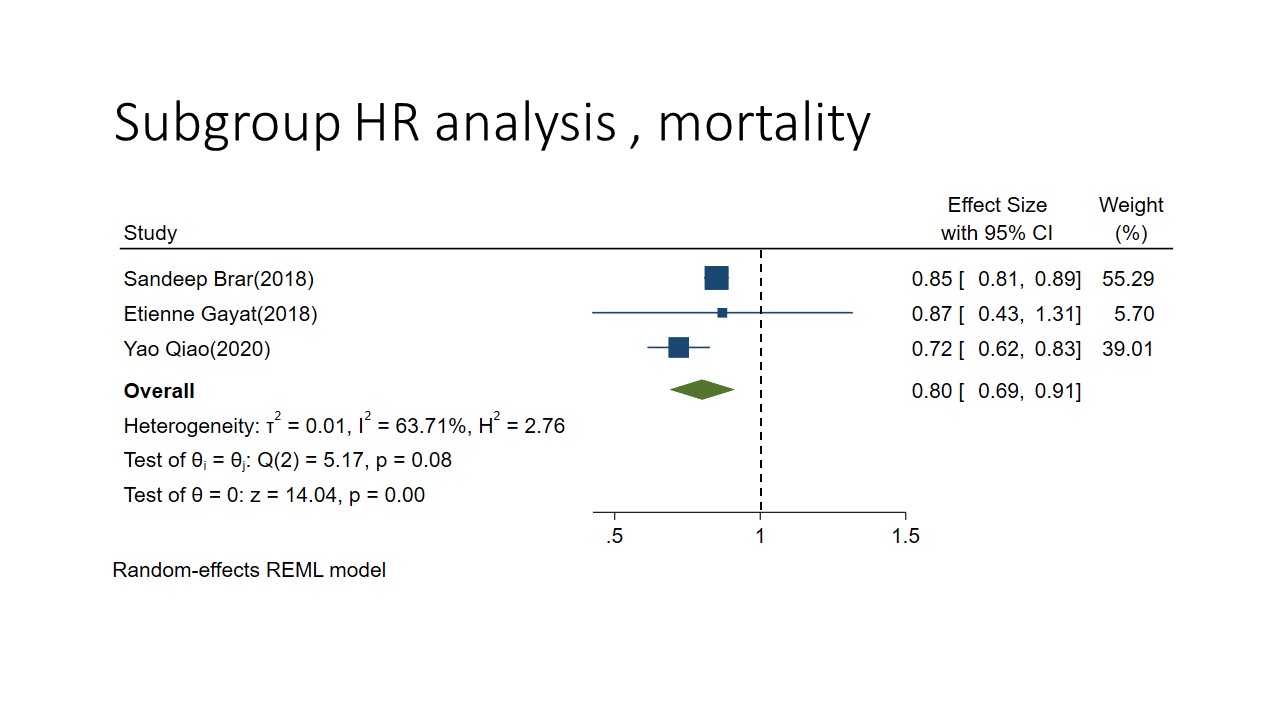


**Supplemental Figure 7.** Forest plot showing all-cause mortality according to ACEi/ARB use after AKI by pooling the multivariate adjusted odds ratios.

1. **Summary of contextual factor data**

In our study, 7 case control studies with 70,801 participants were included in this meta-analysis. In Brar et al., 22,193 cases were included with ACEi/ARB administration after hospital discharge within the first 6 months and 15% decrease in the all-cause mortality rate was noted in patients with AKI after 2-year follow-up period (HR, 0.85; 95% CI, 0.81–0.89). Gayat et al. included 611 AKI patients including KDIGO stage 1 to 3 who received ACEi/ARB at ICU discharge with lower 1-year mortality rates including unadjusted, adjusted and propensity-matched score (p<0.001). Qiao et al. included 3,909 patients initiating ACEi/ARB prescription with experiencing an eGFR decrease to below 30 mL/min/1.73 m2, and 1,235 patients discontinued ACEi/ARB usage within 6 months after the eGFR decrease. Patients who discontinued ACEi/ARB treatment had a higher risk of mortality (HR, 1.39; 95% CI, 1.20-1.60) and MACE (HR, 1.37; 95% CI, 1.20-1.56). Bidulka et al. conducted two parallel studies enrolling 7,303 patients in England and 1,790 ones in Sweden, and the risk of death increased in those of discontinuation of ACEi/ARB (HR 1.27; 95% CI 1.15–1.41) in England, but not in Sweden.

In addition to mortality, other outcomes including recurrent/worsening kidney events, renal recovery, heart failure and stroke have also been discussed. Hsu et al. included 10,242 AKI patients who newly received ACEi/ARB and showed that exposure to ACEi/ARB was not associated with higher incidence of recurrent/worsening kidney events AKI (aOR, 0.71; 95% CI, 0.45 to 1.12) in marginal structural causal inference models after baseline and potential time-dependent confounders adjustment. In Hines et al., 345 AKI survivors were included, and there was no difference in the rate of AKD patients with and without ACEi/ARB usage when discharge (12.5 vs. 15.0%, p = 0.530). This implied the exposure to ACEi/ARB before or during AKI was not associated with AKD and the renal recovery after AKI. In Bidulka et al., the risk of heart failure and AKI did not increase between the groups of ACEi/ARB continuation or not in English and Swedish cohort, but stroke was less common in patients with discontinuation of ACEi/ARB treatment in Sweden (HR 0.56; 95% CI 0.34–0.93).

In Scarton et al, the AKIKI trial included 348 ICU patients with KDIGO stage 3 who discharged alive from ICU, and 2-year all-cause mortality did not have significant difference between ACEi/ARB users and non-users (p = 0.18).

From most of the references above, the usage of ACEi/ARB in AKI patients and the continuation of the ACEi/ARB use in AKI status improved the mortality and did not increase the risk of other outcomes, including recurrent or worsening kidney events, AKD, heart failure and stroke. However, according to The Kidney Disease: Improving Global Outcomes (KDIGO) Conference consensus, ACEi/ARBs discontinuation was suggested during AKI, and the latest AKI consensus report of the Acute Disease Quality Initiative (ADQI) raised the recommendations relating to the ACEi/ARB treatment of patients in AKD, however, the beneficial impact of ACEi/ARBs on AKD stage is not illuminated since there is no large randomized controlled trials (RCTs) available on ACEi/ARB continuation versus discontinuation in AKI patients during hospitalization.

Of note, our data revealed ACEi/ARB users had lower risk of all-cause mortality and recurrent/worsening kidney events compared to non-users. Hyperkalemia should be cautious during the usage of medication. Though the literatures were highly heterogeneous, the funnel meta-regression analysis revealed limited publication bias. And this study is the most comprehensive systemic review including the largest population of post-AKI patients receiving ACEi/ARB treatment. However, there are several limitations in our study, including the different illness severity in each study that might regard to other factors of mortality, the lack of baseline renal function and the heterogeneity of AKI staging, and the drug indications among the usage of ACEi/ARB which eliminated by adjusted odds ratio analysis with variable subgroup analysis in our analysis, and potential unmeasured confounding interactions in these observation-designed studies. Most importantly, the timing of initiation of ACEi/ARB usage after AKI episodes might lead future RCTs design to evaluate the optimal timing of initiation of ACEi/ARB usage in AKI patients.

In conclusion, treatment with ACEi/ARB in patients after AKI can reduce all-cause mortality and recurrent kidney events. Continuation of ACEi/ARB treatment in AKI patient may be beneficial in survival. We also raised the issue that hyperkalemia should be cautious after ACEi/ARB prescription. As to our knowledge, this systematic review raised the best available evidence for current interim guidance.

1. **PROSPERO protocol registration**

1. * Review title.

Give the title of the review in English

The effect of Renin Angiotensin Aldosterone System inhibitor major kidney outcomes after acute kidney injury: a systemic review and meta-analysis

2. Original language title.

For reviews in languages other than English, give the title in the original language. This will be displayed with the English language title.

The effect of Renin Angiotensin Aldosterone System inhibitor major kidney outcomes after acute kidney injury: a systemic review and meta-analysis

3. * Anticipated or actual start date.

Give the date the systematic review started or is expected to start.

18/08/2020

4. * Anticipated completion date.

Give the date by which the review is expected to be completed.

30/11/2020

5. * Stage of review at time of this submission.

Tick the boxes to show which review tasks have been started and which have been completed. Update this field each time any amendments are made to a published record.

**Reviews that have started data extraction (at the time of initial submission) are not eligible for**

**inclusion in PROSPERO**. If there is later evidence that incorrect status and/or completion date has been supplied, the published PROSPERO record will be marked as retracted.

This field uses answers to initial screening questions. It cannot be edited until after registration.

The review has not yet started: No

**Review stage Started Completed**

Preliminary searches Yes Yes

Piloting of the study selection process Yes Yes

Formal screening of search results against eligibility criteria Yes No

Data extraction No No

Risk of bias (quality) assessment Yes No

Data analysis Yes No

Provide any other relevant information about the stage of the review here.

6. * Named contact.

The named contact is the guarantor for the accuracy of the information in the register record. This may be any member of the review team.

Jui-Yi Chen

Email salutation (e.g. "Dr Smith" or "Joanne") for correspondence:

Mr Chen

7. * Named contact email.

Give the electronic email address of the named contact.

[a50601@mail.chimei.org.tw](mailto:a50601@mail.chimei.org.tw)

8. Named contact address

Give the full institutional/organisational postal address for the named contact.

No. 901, Zhonghua Rd., Yongkang Dist., Tainan City 710, Taiwan (R.O.C.)

9. Named contact phone number.

Give the telephone number for the named contact, including international dialling code.

886-917652658

10. * Organisational affiliation of the review.

Full title of the organisational affiliations for this review and website address if available. This field may be completed as 'None' if the review is not affiliated to any organisation.

Division of Nephrology, Department of Internal Medicine, Chi Mei Medical Center, Tainan, Taiwan

Organisation web address:

<http://www.chimei.org.tw/main/cmh_department/54220/english/>

11. * Review team members and their organisational affiliations.

Give the personal details and the organisational affiliations of each member of the review team. Affiliation refers to groups or organisations to which review team members belong. **NOTE: email and country now** **MUST be entered for each person, unless you are amending a published record.**

Mr Jui-Yi Chen. Division of Nephrology, Department of Internal Medicine, Chi Mei Medical Center, Tainan,

Taiwan

12. * Funding sources/sponsors.

Details of the individuals, organizations, groups, companies or other legal entities who have funded or sponsored the review.

nil

Grant number(s)

State the funder, grant or award number and the date of award

Nil

13. * Conflicts of interest.

List actual or perceived conflicts of interest (financial or academic).

None

14. Collaborators.

Give the name and affiliation of any individuals or organisations who are working on the review but who are not listed as review team members. **NOTE: email and country must be completed for each person,** **unless you are amending a published record.**

Dr VIN-CENT WU. National Taiwan University Hospital: Taipei, TW

15. * Review question.

State the review question(s) clearly and precisely. It may be appropriate to break very broad questions down

into a series of related more specific questions. Questions may be framed or refined using PI(E)COS or similar where relevant.

Continuing Renin Angiotensin Aldosterone System inhibitor after kidney injury will worsen the renal function?

16. * Searches.

State the sources that will be searched (e.g. Medline). Give the search dates, and any restrictions (e.g.language or publication date). Do NOT enter the full search strategy (it may be provided as a link or attachment below.)

17. URL to search strategy.

Upload a file with your search strategy, or an example of a search strategy for a specific database, (including the keywords) in pdf or word format. In doing so you are consenting to the file being made publicly accessible. Or provide a URL or link to the strategy. Do NOT provide links to your search **results**.

https://www.crd.york.ac.uk/PROSPEROFILES/204885_STRATEGY_20200826.pdf

Alternatively, upload your search strategy to CRD in pdf format. Please note that by doing so you are consenting to the file being made publicly accessible.

Do not make this file publicly available until the review is complete

18. * Condition or domain being studied.

Give a short description of the disease, condition or healthcare domain being studied in your systematic review.

Acute kidney injury, Renin Angiotensin Aldosterone System inhibitor

19. * Participants/population.

Specify the participants or populations being studied in the review. The preferred format includes details of both inclusion and exclusion criteria.

IEnxcclulussioionn: :Aduolltess aceftnetrs a(ucnudtee rk i1d8n eyye ainrsju oryf age)

20. * Intervention(s), exposure(s).

Give full and clear descriptions or definitions of the interventions or the exposures to be reviewed. The preferred format includes details of both inclusion and exclusion criteria.

The ACEi group are defined as those keep using Renin Angiotensin Aldosterone System inhibitor after acute kidney injury

21. * Comparator(s)/control.

Where relevant, give details of the alternatives against which the intervention/exposure will be compared (e.g. another intervention or a non-exposed control group). The preferred format includes details of both inclusion and exclusion criteria.

The control group are defined as those didn't use Renin Angiotensin Aldosterone System inhibitor after acute kidney injury

22. * Types of study to be included.

Give details of the study designs (e.g. RCT) that are eligible for inclusion in the review. The preferred format includes both inclusion and exclusion criteria. If there are no restrictions on the types of study, this should be stated.

We will included prospective cohort study, retrospective cohort studies, and observational study for the assessment of recurrent acute kidney injury.

23. Context.

Give summary details of the setting or other relevant characteristics, which help define the inclusion or exclusion criteria.

Studies for those had ever acute kidney injury

24. * Main outcome(s).

Give the pre-specified main (most important) outcomes of the review, including details of how the outcome is defined and measured and when these measurement are made, if these are part of the review inclusion criteria.

recurrent acute kidney injury during follow-up

* Measures of effect

Please specify the effect measure(s) for you main outcome(s) e.g. relative risks, odds ratios, risk difference, and/or 'number needed to treat.

25. * Additional outcome(s).

List the pre-specified additional outcomes of the review, with a similar level of detail to that required for main outcomes. Where there are no additional outcomes please state ‘None’ or ‘Not applicable’ as appropriate to the review

Mortality during follow up

* Measures of effect

Please specify the effect measure(s) for you additional outcome(s) e.g. relative risks, odds ratios, risk difference, and/or 'number needed to treat.

odds ratio

26. * Data extraction (selection and coding).

Describe how studies will be selected for inclusion. State what data will be extracted or obtained. State how this will be done and recorded.

The following data were extracted from the full-text articles: the first author name, year of publication, sample size, study design, patient inclusion criteria, patient demographics, clinical outcome and adverse events.

27. * Risk of bias (quality) assessment.

State which characteristics of the studies will be assessed and/or any formal risk of bias/quality assessment tools that will be used.

Outcome about renal event of acute kidney injury

IAf pthperareis awla tsh ed isstaugdriees muesinntgs Tbheetw Neewn ctwasot lree-vOiettwaweras ,S tchairlde abuyt htwoor jruedvgiewd ethrse inclusion of the article

28. * Strategy for data synthesis.

Describe the methods you plan to use to synthesise data. This **must not be generic text** but should be **specific to your review** and describe how the proposed approach will be applied to your data. If metaanalysis is planned, describe the models to be used, methods to explore statistical heterogeneity, and software package to be used.

odds ratio for recurrent acute kidney injury and mortality will be combined using fix effects meta-analysis

29. * Analysis of subgroups or subsets.

State any planned investigation of ‘subgroups’. Be clear and specific about which type of study or

participant will be included in each group or covariate investigated. State the planned analytic approach.

common variables including age, DM, hypertension, congestive heart failure for meta-regression

30. * Type and method of review.

Select the type of review, review method and health area from the lists below.

Type of review

Cost effectiveness: No

Diagnostic: No

Epidemiologic: No

Individual patient data (IPD) meta-analysis: Yes

Intervention: Yes

Meta-analysis: Yes

Methodology: No

Narrative synthesis: No

Network meta-analysis: No

Pre-clinical: No

Prevention: No

Prognostic: No

Prospective meta-analysis (PMA): No

Review of reviews: No

Service delivery: No

Synthesis of qualitative studies: No

Systematic review: Yes

Other: No

Health area of the review

Alcohol/substance misuse/abuse: No

Blood and immune system: No

Cancer: No

Cardiovascular: No

Care of the elderly: No

Child health: No

Complementary therapies: No

COVID-19: No

Crime and justice: No

Dental: No

Digestive system: No

Ear, nose and throat: No

Education: No

Endocrine and metabolic disorders: No

Eye disorders: No

General interest: No

Genetics: No

Health inequalities/health equity: No

Infections and infestations: No

International development: No

Mental health and behavioural conditions: No

Musculoskeletal: No

Neurological: No

Nursing: No

Obstetrics and gynaecology: No

Oral health: No

Palliative care: No

Perioperative care: No

Physiotherapy: No

Pregnancy and childbirth: No

Public health (including social determinants of health): No

Rehabilitation: No

Respiratory disorders: No

Service delivery: No

Skin disorders: No

Social care: No

Surgery: No

Tropical Medicine: No

Urological: No

Wounds, injuries and accidents: No

Violence and abuse: No

31. Language.

Select each language individually to add it to the list below, use the bin icon to remove any added in error.

English

There is not an English language summary

32. * Country.

Select the country in which the review is being carried out. For multi-national collaborations select all the countries involved.

Taiwan

33. Other registration details.

Name any other organisation where the systematic review title or protocol is registered (e.g. Campbell, or The Joanna Briggs Institute) together with any unique identification number assigned by them. If extracted data will be stored and made available through a repository such as the Systematic Review Data Repository(SRDR), details and a link should be included here. If none, leave blank.

34. Reference and/or URL for published protocol.

If the protocol for this review is published provide details (authors, title and journal details, preferably in Vancouver format)

Add web link to the published protocol.

Or, upload your published protocol here in pdf format. Note that the upload will be publicly accessible.

No I do not make this file publicly available until the review is complete

Please note that the information required in the PROSPERO registration form must be completed in full even if access to a protocol is given.

35. Dissemination plans.

Do you intend to publish the review on completion?

No

Give brief details of plans for communicating review findings.?

36. Keywords.

Give words or phrases that best describe the review. Separate keywords with a semicolon or new line.

Keywords help PROSPERO users find your review (keywords do not appear in the public record but are included in searches). Be as specific and precise as possible. Avoid acronyms and abbreviations unless these are in wide use.

acute kidney injury; Renin Angiotensin Aldosterone System inhibitor;angiotensin- converting enzyme inhibitors

37. Details of any existing review of the same topic by the same authors.

If you are registering an update of an existing review give details of the earlier versions and include a full bibliographic reference, if available.

38. * Current review status.

Update review status when the review is completed and when it is published.New registrations must be ongoing.

Please provide anticipated publication date

Review_Ongoing

39. Any additional information.

Provide any other information relevant to the registration of this review.

40. Details of final report/publication(s) or preprints if available.

Leave empty until publication details are available OR you have a link to a preprint. List authors, title and journal details preferably in Vancouver format.

Give the link to the published review or preprint.

1. **The GRADE results**

**
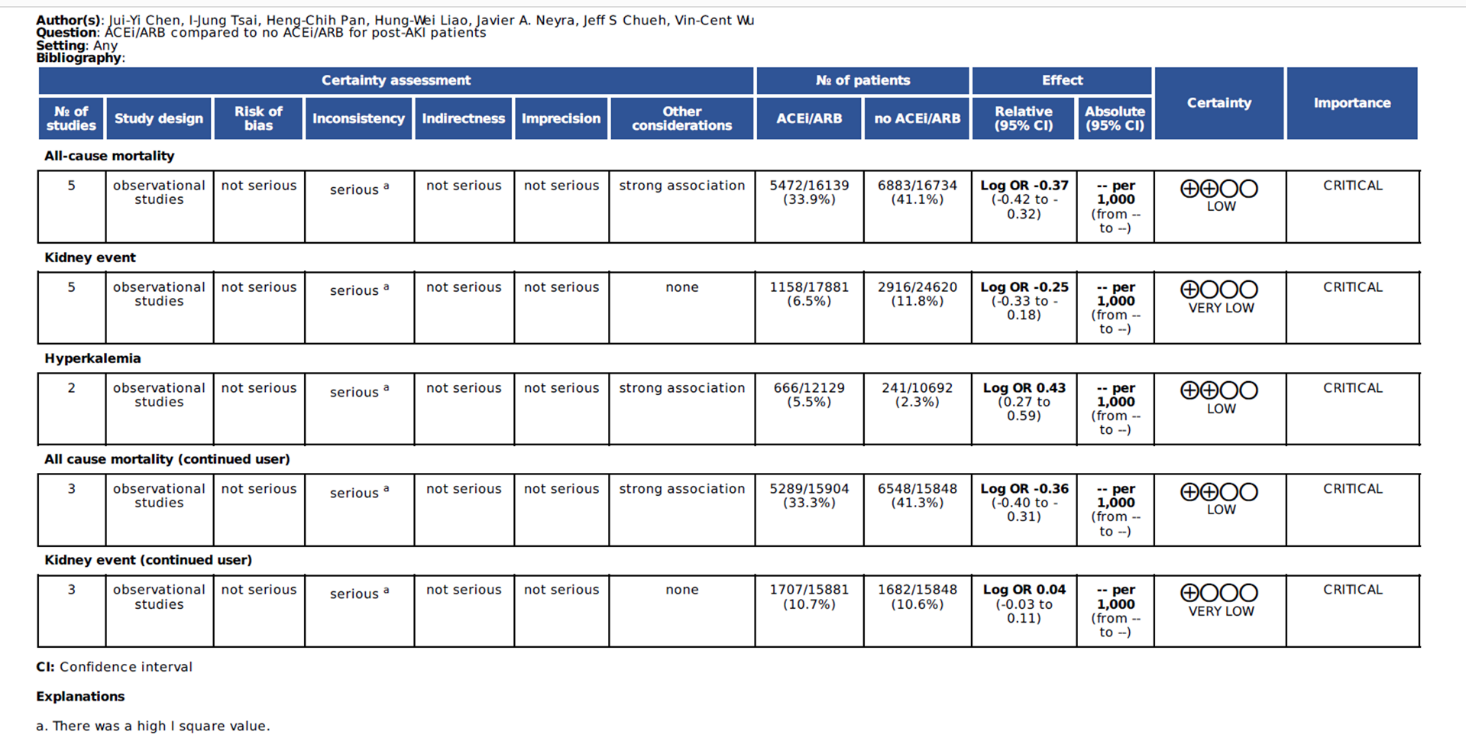
**

1. **Flow chart showing Search strategy for studies in China.
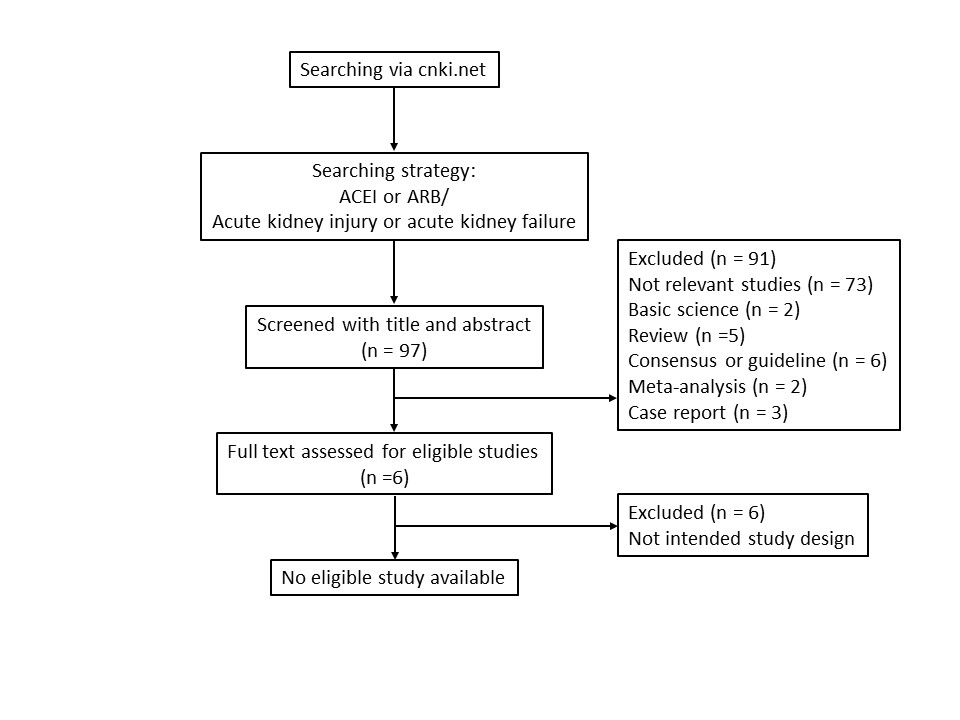
**
